# Dynamic DNA methylation changes reveal tissue-specific gene expression in sugarcane

**DOI:** 10.3389/fpls.2022.1036764

**Published:** 2022-10-13

**Authors:** Yajie Xue, Chengwu Zou, Chao Zhang, Hang Yu, Baoshan Chen, Haifeng Wang

**Affiliations:** ^1^ State Key Laboratory for Conservation and Utilization of Subtropical Agro-Bioresources, College of Agriculture, Guangxi University, Nanning, China; ^2^ Guangxi Colleges and Universities Key Laboratory of Crop Cultivation and Tillage, Guangxi University, Nanning, China

**Keywords:** sugarcane, DNA methylation, differentially methylated regions, DNA methylation valleys, epigenetics

## Abstract

DNA methylation is an important mechanism for the dynamic regulation of gene expression and silencing of transposons during plant developmental processes. Here, we analyzed genome-wide methylation patterns in sugarcane (*Saccharum officinarum*) leaves, roots, rinds, and piths at single-base resolution. DNA methylation patterns were similar among the different sugarcane tissues, whereas DNA methylation levels differed. We also found that DNA methylation in different genic regions or sequence contexts plays different roles in gene expression. Differences in methylation among tissues resulted in many differentially methylated regions (DMRs) between tissues, particularly CHH DMRs. Genes overlapping with DMRs tended to be differentially expressed (DEGs) between tissues, and these DMR-associated DEGs were enriched in biological pathways related to tissue function, such as photosynthesis, sucrose synthesis, stress response, transport, and metabolism. Moreover, we observed many DNA methylation valleys (DMVs), which always overlapped with transcription factors (TFs) and sucrose-related genes, such as *WRKY*, *bZIP*, *WOX*, *SPS*, and *FBPase*. Collectively, these findings provide significant insights into the complicated interplay between DNA methylation and gene expression and shed light on the epigenetic regulation of sucrose-related genes in sugarcane.

## Introduction

DNA methylation is among the most common epigenetic modifications in eukaryotic genomes and is involved in regulating gene transcription and transposon silencing (Law and Jacobsen, 2010; Zhang et al., 2018a). In animals, DNA methylation mainly occurs at CG sites, whereas in plants, it occurs at CG, CHG, and CHH sites (H represents A, T, or C) (Law and Jacobsen, 2010). Information on DNA methylation in plants is mainly derived from model plants, such as *Arabidopsis thaliana* and rice (*Oryza sativa*). Methylation in three different contexts is established and maintained by different pathways. CG methylation is mainly catalyzed by methyltransferase 1 (MET1) (Kankel et al., 2003), while chromomethylase 3 (CMT3) is responsible for maintaining CHG methylation. Recent studies have shown that CMT2 is also involved in the maintenance of CHG methylation (Lindroth et al., 2001; Stroud et al., 2014) and plays a major role in maintaining asymmetric CHH methylation. CHH methylation maintained by CMT2 always occurs at long transposable elements (TEs), which are often located in peri-centromeric regions (Zemach et al., 2013; Stroud et al., 2014; Gouil and Baulcombe, 2016). In all three contexts, cytosines can be *de novo* methylated by the RNA-directed DNA methylation (RdDM) pathway, which also involves domains rearranged methyltransferase (DRM2) and several other proteins (Law and Jacobsen, 2010; Kawashima and Berger, 2014; Cuerda-Gil and Slotkin, 2016). DNA methylation is dynamically regulated by methylases and demethylases, and four DNA demethylases have been identified in *A. thaliana*, including ROS1, DME, DML2, and DML3 (Choi et al., 2002; Gong et al., 2002; Morales-Ruiz et al., 2006; Ortega-Galisteo et al., 2008).

Recently, extensive studies have shown that DNA methylation plays an important role in plant growth, development, and stress response (Zhang et al., 2018a; Chang et al., 2020). For example, deficient non-CG methylation levels in Arabidopsis resulted in a twisted leaf shape, shorter stature, and partial sterility phenotypic defects (Chan et al., 2006). In addition, 70% of drought-induced methylation changes in rice were recovered after irrigation resumed (Wang et al., 2011). Salt stress inhibits DNA methylation in the promoter region of *OsMYB91*, promoting its expression and increasing salt tolerance in rice (Zhu et al., 2015). Although extensive studies on plant DNA methylation have been reported, most have focused on models or economically important crops, such as rice, soybean (*Glycine max*), sorghum (*Sorghum bicolor*), cassava (*Manihot esculenta*), and tomato (*Solanum lycopersicum*) (Li et al., 2012; Song et al., 2013; Wang et al., 2015; Turco et al., 2017; Wang et al., 2018). These genomes are relatively small and have low complexity, and very few DNA methylation studies have been conducted on species with large genomes and high genome complexity, such as bread wheat (*Triticum aestivum*), Norway spruce (*Picea abies*), and Chinese pine (*Pinus tabuliformis*) (Ausin et al., 2016; Li et al., 2019; Niu et al., 2022). Owing to the complexity of the sugarcane (*Saccharum officinarum*) genome (large genome size and polyploidy), its reference genome has only recently been released, providing an unprecedented opportunity to investigate the role of DNA methylation in sugarcane growth.

Here, we explored genome-wide DNA methylation profiles in four different sugarcane tissues using whole-genome bisulfite sequencing (WGBS). Combined with transcriptome data, we investigated the association between DNA methylation changes and expression divergence among four tissues (leaf, rind, pith, and root). Moreover, comparative multi-omics analysis revealed the regulatory role of DNA methylation variation in the different sugarcane tissues, especially in genes related to important agronomic traits. Thus, our study provides a unique insight into the role of DNA methylation in sugarcane research.

## Materials and methods

### Plant materials and tissue collection

Sugarcane cultivar Zhongzhe No. 1 was grown at the Fusui planting base of Guangxi University (22°17’N, 107°31’E). We selected sugarcane at the mature stage for sampling, in which root, leaf +1, rind and pith of 10^th^ stalk were collected.

### Whole-genome bisulfite sequencing and analysis

The whole-genome bisulfite sequencing (WGBS) library was constructed as described by Wang (Wang et al., 2015). WGBS libraries were sequenced on an Illumina NovaSeq 6000 system (Illumina, San Diego, CA, USA) to obtain pair-end 150-bp reads.

Raw 150-bp paired-end reads were subjected to quality control filters using FASTQC (http://www.bioinformatics.babraham.ac.uk/projects/fastqc/) and trimmed using Trimmomatic v0.39 (Bolger et al., 2014). The clean reads were aligned to the sugarcane reference genome (Zhang et al., 2018b) using BSMAP v2.90 (Xi and Li, 2009), and up to 10 base mismatches were allowed. Only uniquely mapped reads were used to estimate the methylation ratios. The methylation ratio was calculated from the number of sequenced cytosines divided by the total read depth [mC/(mC + non-mC)], and visual analysis was conducted using ViewBS v0.1.9 (Huang et al., 2018).

Reproducibility between replicates of BS-seq was calculated as methylation levels in 100-kb regions in both replicates, and Pearson correlation coefficients between replicates were calculated.

The differentially methylated regions (DMRs) between different tissues were calculated using the methylKit R package (Akalin et al., 2012); the genome was divided into 100bp bins and mC sites covered by more than 3 reads were used for subsequent analysis. The methylation differences between all sequence contexts were as follows: CG difference was greater than 0.4, CHG difference was greater than 0.2, and CHH difference was greater than 0.1.

### Transcriptome sequencing and analysis

Total RNA was isolated from the same tissues used in the WGBS library using TRIzol reagent (Invitrogen, Carlsbad, CA, USA) according to the manufacturer’s instructions. The RNA-seq library was constructed following the Illumina kit’s recommendation and sequenced using Illumina NovaSeq 6000 (Illumina) with paired-end reads of 150 bp.

FASTQC was used for initial read quality control. Clean reads were mapped to the sugarcane reference genome (Zhang et al., 2018b) using hisat2 V2.1.0 with default settings (Kim et al., 2015). We used Stringtie v2.1.4 to calculate the gene expression levels (Pertea et al., 2015). Differentially expressed genes (DEGs) were identified using DESeq2 v1.32.0 (Sahraeian et al., 2017) with a 4-fold change and FDR < 0.05.

### Gene ontology enrichment analysis

Gene functions were annotated using eggNOG-mapper (Huerta-Cepas et al., 2019), and Gene Ontology (GO) enrichment analysis was performed using GOATOOLS with false-discovery rate correction (<0.05) (Klopfenstein et al., 2018).

### Identification and characterization of sugarcane DMVs

The DNA methylation valleys (DMVs) in sugarcane were identified as previously described (Lin et al., 2017; Chen et al., 2018; Li et al., 2018). Briefly, the genome was first divided into 1-kb bins, and we calculated the DNA methylation levels in each bin. The DMV is the bin where the methylation levels of all sequence contexts are less than 5% in all tissues. Next, all overlapping DMVs were merged (**Figure 6B**). Finally, genes (gene body and flanking 1 kb) located in the DMVs were defined as DMV genes.

## Results

### Characterization of DNA methylation patterns among different sugarcane tissues

To investigate the DNA methylation patterns in sugarcane, we used WGBS to examine cytosine methylation in four sugarcane tissues: leaf, root, rind, and pith. Each sample was sequenced in two biological replicates, and approximately 70% of the reads were aligned to the reference genome, except for one biological replicate of the roots (**Table S1**). Pearson’s correlation coefficients between different biological replicates were greater than 0.95, except in the roots, indicating the high reproducibility and accuracy of our sequencing data (**Figure S1**). Next, we merged the two replicates because their data were highly correlated. There were 1,137 million cytosines that could be methylated in sugarcane, accounting for 39.2% of the sugarcane genome; approximately 87% of the total cytosines were covered by at least one read (**Figure S2**).

**Figure 1:**
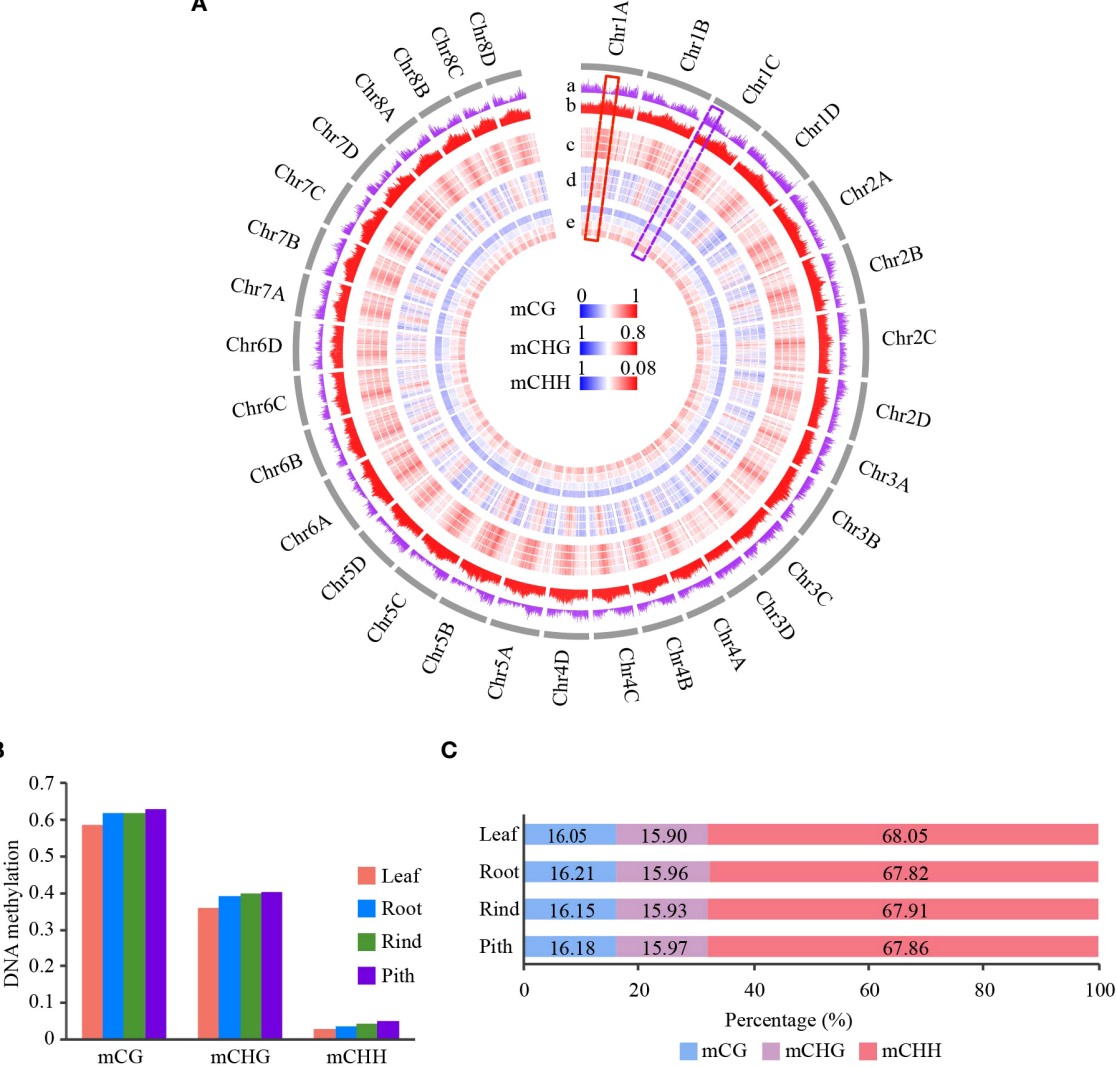
Genome-wide DNA methylation profile of different tissues in sugarcane. **(A)** Circle plot of gene and TE densities and methylation level of CG, CHG, and CHH across 32 homologous chromosomes in sugarcane. DNA methylation level is represented in a heatmap; blue and red indicate low and high methylation levels, respectively. Gene and TE density are represented in a histogram. Average DNA methylation level and gene/TE density are calculated using a 500-kb window. The gray circle indicates chromosomes. From the outer circle to the inner circle: a, gene density; b, TE density (TE density is the ratio of TE length to window length); c, CG methylation; d, CHG methylation; e, CHH methylation. For the DNA methylation circle, the order from outer to inner is Leaf, Root, Rind, and Pith. **(B)** Global average DNA methylation levels of CG, CHG, and CHH across different tissues in sugarcane. **(C)** Relative proportion of methyl-cytosines in the three sequence contexts across different tissues.

From the distribution of global DNA methylation, we found that gene-enriched regions showed low CG and CHG methylation levels, while transposable element (TE)-enriched regions had high methylation levels (**Figure 1A**). This result is consistent with previous studies on other plants (Song et al., 2013; Wang et al., 2015; Song et al., 2015). In addition, we found that CHH methylation was slightly enriched in the gene-enriched regions compared with that in regions with dense CG and CHG methylation (**Figure 1A**). The distribution of CHH methylation in sugarcane is consistent with that in maize (*Zea mays*) (Gent et al., 2013). We also found a negative correlation between gene and TE densities (*R*=-0.68, *p* < 2.2e-16) (**Figures 1A** and **Figure S3**). To better understand the relationship between DNA methylation levels and gene and TE densities, we calculated their correlation coefficients. We found that both mCG and mCHG methylation negatively correlated with gene density, indicating that these two DNA methylation contexts were mostly located in gene-poor heterochromatic regions. However, leaf tissue showed no correlation, and the other three tissues showed positive correlations between gene density and mCHH levels (**Figure S4**). This result was consistent with findings in rice, sorghum (*Sorghum bicolor*), and maize (Gent et al., 2013; Niederhuth et al., 2016). As expected, TE density positively correlated with CG and CHG methylation levels (*R* > 0.7) but showed a weak correlation with CHH methylation (|R|<0.3) (**Figure S5**).

To investigate the relationship between TE methylation and the distance between TEs and adjacent genes, we calculated the methylation levels of TEs. We found that higher TE CHH methylation levels in all tissues positively correlated with the closer distances of TEs to the gene, but this phenomenon was not observed in CG and CHG methylation (**Figure S6**). Altogether, these results suggest that gene and transposon densities and methylation levels correlate, and the distribution of genes and transposons in the genome jointly shapes the landscape of DNA methylation in different regions of the genome.

Genome-wide distribution and global DNA methylation levels showed obvious DNA methylation changes among the four tissues (**Figures 1A, B**). Pith tissue showed the highest DNA methylation levels, followed by the rind, root, and leaf. In contrast to methylation levels, we found no significant differences in the proportion of methylcytosines among the four tissues, with CHH methylcytosine being the most abundant (>67%), followed by CG and CHG methylcytosines (**Figure 1C**). This finding is consistent with other plant studies (Wang et al., 2015; Xu et al., 2018).

### DNA methylation patterns of gene and TE regions

**Figure 2:**
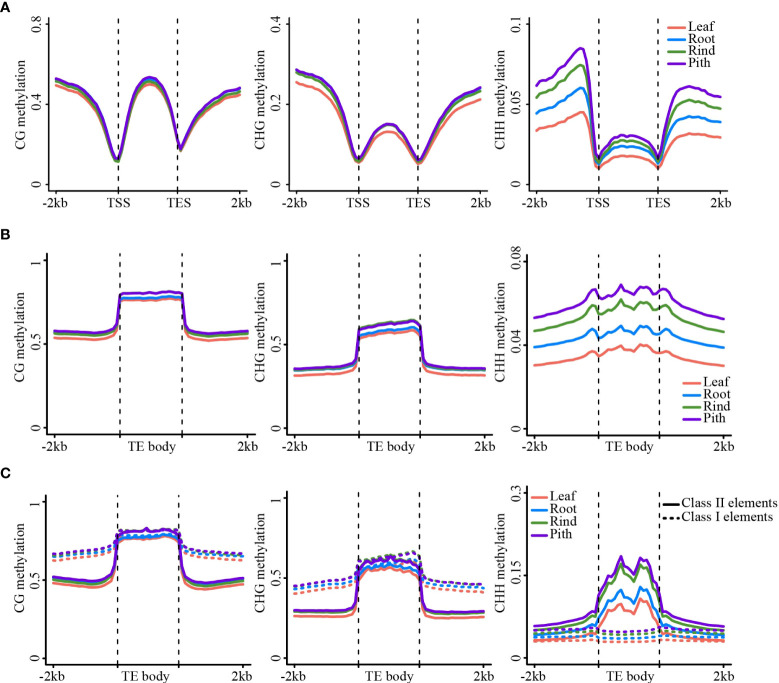
DNA methylation patterns in gene/TE and flanking regions. **(A)** The metaplot of the gene body and the flanking region. **(B)** The metaplot of TE and the flanking region. **(C)** The metaplot of the Class II TE and Class I TE. CG (left), CHG (middle), CHH (right).

Genome-wide DNA methylation analysis revealed substantial differences in methylation levels among the four sugarcane tissues. Next, we analyzed DNA methylation levels in the gene and transposon regions of the four tissues. The results of the meta-analysis of gene and TE regions were consistent with those of the genome-wide methylation analysis, i.e., leaf and pith tissues showed the lowest and highest methylation levels, respectively (**Figures 2A, B**). This trend was also consistent between the gene body and TE regions (**Figure 2**). Strikingly, gene body regions showed relatively high CHG and CHH methylation levels, in addition to dense CG methylation (**Figure 2A**), differing from many other plant species, such as Arabidopsis and rice (Cokus et al., 2008; Li et al., 2012). In the sugarcane genome, more than 58.7% of the sequences consisted of repetitive elements (Zhang et al., 2018b), such as TEs, and 42.3% of protein-coding genes contained TE sequences in the gene body regions, particularly in intron regions (**Figure S7A**). After excluding genes with intronic TE insertions, we found that the methylation levels of gene body regions were notably reduced in all three sequence contexts, especially non-CG methylation levels. However, methylation levels of the flanking regions were slightly reduced (**Figures S7B, C**). This result suggested that most of the non-CG methylation of gene body regions was determined by intronic TE insertions, which have been reported in maize and other plant genomes with abundant TEs (Wang et al., 2015; Wang et al., 2021; Niu et al., 2022).

Next, we compared DNA methylation between different types of transposons, including Class I and II transposons. Class I transposons showed higher levels of CG and CHG methylation than Class II transposons, both in the transposon body and flanking regions. However, CHH methylation was higher in Class II transposons than that in Class I transposons (**Figure 2C**). Long terminal repeat (LTR)-type transposons mainly include Gypsy and Copia LTRs, whereas DNA transposons contain several types of transposons (**Figure S8A**). The Gypsy and Copia LTRs showed very similar DNA methylation patterns (**Figures S8B–E**). However, different types of DNA transposons exhibit substantially different methylation patterns. For example, the CHH methylation level of the PIF-Harbinger transposon was significantly higher than that of the other types of transposons (**Figure S9**).

### Active demethylase is associated with reduced DNA methylation among different tissues

**Figure 3:**
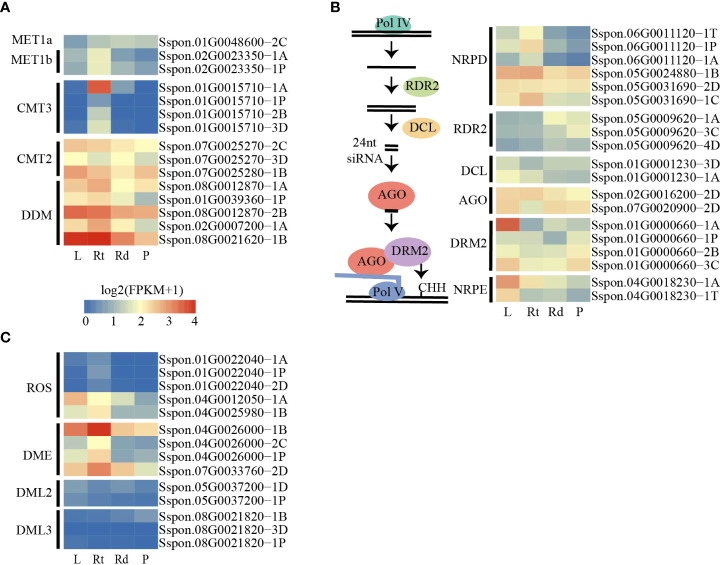
DNA methylated and demethylated genes are active in sugarcane tissues. **(A)** Heatmap showing the expression patterns of methylation-related genes among tissues. **(B)** Schematic diagram of the canonical RdDM pathway (left). Heatmap showing the expression patterns of canonical RdDM pathway genes among tissues. **(C)** Heatmap showing the expression patterns of demethylated genes. L, leaf; Rt, root; Rd, rind; P, pith.

DNA methylation levels are dynamically regulated by DNA methylases and demethylases. The decrease in DNA methylation levels can be attributed to the low expression of DNA methylase or high demethylase expression. To investigate the changes in DNA methylation levels among the four tissues, we searched for and annotated the DNA methylase and demethylase genes in the sugarcane genome (**Table S2**). As the sugarcane genome was assembled and annotated into four sets of homologous chromosomes, we identified more homologous genes in the sugarcane genome than in Arabidopsis and other plants. We first examined the expression levels of DNA methylase across the four tissues, and we did not observe a gradual increase in expression levels of these genes from the leaves to the roots and stem (rinds and piths) (**Figures 3A, B**). In addition, we found that only a few genes were differentially expressed in the RdDM pathway (**Figures 3B**). These results suggest that increased DNA methylation levels from the leaves to the piths were not attributed to the increased expression of DNA methylases and genes involved in the RdDM pathway.

We also examined the expression of putative DNA demethylase genes. Consistent with the changes in DNA methylation levels across the four tissues, we found that several demethylated genes, such as *ROS* and *DME*, were expressed at lower levels in pith tissue than in other tissues (**Figure 3C**). This result suggests that the DNA demethylation pathway plays a critical role in methylation level changes across the four tissues.

### The association between DNA methylation and gene activity

**Figure 4:**
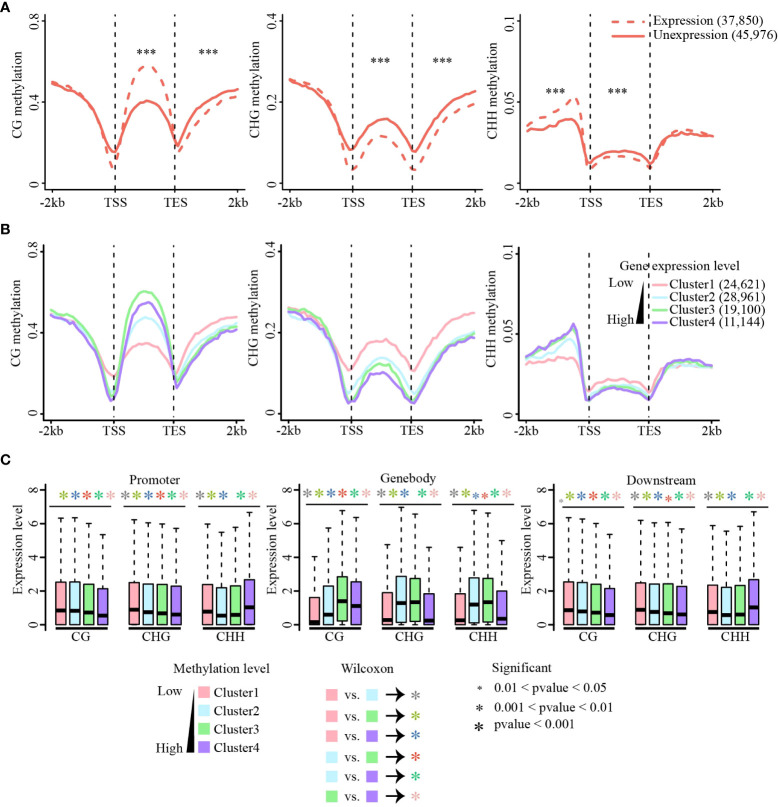
Association of methylation and gene expression. **(A)** Methylation level changes between the expressed and unexpressed genes in CG, CHG, and CHH sequence contexts. Methylation differences between expressed and unexpressed genes were tested using the Wilcoxon rank-sum test. ***p-value < 0.001. **(B)** Correlations between methylation levels (CG, CHG, and CHH) and gene expression across gene body and flanking regions. Methylation level of each gene group [Cluster1 (FPKM = 0), Cluster2 (0 < FPKM ≤ 2), Cluster3 (2 < FPKM ≤ 10), Cluster4 (FPKM ≥ 10)] were calculated. CG (left), CHG (middle), and CHH (right). **(C)** Expression levels of methylated genes in the gene body and flanking regions. Genes were divided into four quartiles based on methylation levels, from the first quartile (the lowliest methylated 25% of genes) to the fourth quartile (the most highly methylated 25% of genes). Expression differences between different clusters were tested using the Wilcoxon rank-sum test. The colors of the asterisk (*) represent the comparison between different clusters, and the size of the asterisk (*) indicates the criterion of significance. **(A–C)** are data in leaf tissue.

Cumulative evidence has shown that methylation of the gene body and flanking regions is involved in regulating gene expression (Wang et al., 2015; Zhang et al., 2018a; Xu et al., 2018; Wang et al., 2019; Cai et al., 2021). CG methylation of gene body regions is always positively correlated with gene expression, whereas non-CG methylation of gene body regions negatively correlates with gene expression (Wang et al., 2015; Xu et al., 2018; Wang et al., 2019; Cai et al., 2021). In addition, recent studies have shown that CHH methylation of promoter regions could promote adjacent gene expression (Gent et al., 2013; Xu et al., 2018; Cai et al., 2021). To explore the relationship between DNA methylation and gene expression in sugarcane, we first performed RNA-seq of the same tissues for DNA methylation analysis. We found that approximately 75% of clean reads were aligned to the sugarcane genome, and the Pearson correlation coefficients between different biological replicates of RNA-seq were between 0.88 to 0.96 (**Table S3**), indicating the high reproducibility of our RNA-seq data. All genes were divided into expressed (FPKM ≥ 1, 38,750 genes) and unexpressed (FPKM < 1, 45,976 genes) groups and their methylation levels were calculated separately (**Figure 4A**). Compared with unexpressed genes, CG gene body methylation levels of expressed genes were higher than those of unexpressed genes, whereas non-CG methylation was lower in expressed gene body regions than that in unexpressed genes. Consistent with previous studies (Xu et al., 2018; Wang et al., 2019; Cai et al., 2021), DNA methylation levels at the transcription start site (TSS) and transcription end site (TES) were significantly reduced in expressed genes compared with those in unexpressed genes. Additionally, a significant increase in CHH promoter methylation was observed in the expressed genes. A similar phenomenon was observed in the other three tissues (**Figure S10**).

Next, all expressed genes were divided into four groups according to their expression levels from low to high [FPKM = 0 (Cluster1), 0 < FPKM ≤ 2 (Cluster 2), 2 < FPKM ≤ 10 (Cluster 3), and FPKM > 10 (Cluster 4)], and the methylation level of each group of genes was calculated (**Figures 4B** and **Figure S11**). For all three methylation contexts, the methylation levels near TSS and TES sites decreased as the expression level increased, and the methylation level was lowest when the expression was highest. In the gene body regions, CG methylation was positively correlated with gene expression, and the genes with the medium high expression showed the highest CG methylation. This is consistent with the phenomenon observed in studies on many other plants (Wang et al., 2015; Xu et al., 2018; Wang et al., 2019). Non-CG methylation levels were significantly negatively correlated with gene expression, except for CHH methylation in the promoter regions (**Figure 4B**).

To further examine the relationship between gene expression and DNA methylation in different contexts (CG, CHG, and CHH) and genic regions (i.e., upstream, gene body, and downstream regions), we sorted all genes according to their methylation levels from low to high and divided them into four equal groups (Clusters 1 to 4). Consistent with the above analysis, CG methylation of gene body regions promoted gene expression, but CG methylation at either the upstream or downstream regions always inhibited gene expression. CHG and CHH methylation mostly repressed gene expression, except for upstream CHH methylation (**Figures 4C** and **Figure S12**). Collectively, this relationship between DNA methylation and gene expression is conserved in most of the studied plant species. Our findings indicate that DNA methylation is involved in gene expression regulation and DNA methylation of different genic regions and sequence contexts plays different roles in gene expression.

### Extensive changes in gene expression among different tissues in sugarcane

From the above analysis, we found that DNA methylation levels are associated with gene expression in sugarcane. For example, DNA methylation at different genic regions or sequence contexts affects gene expression differently (**Figure 4**). To further explore gene expression changes across different sugarcane tissues, we examined the expression dynamics across different tissues (leaf, root, rind, and pith) and identified 21,460 DEGs between different tissues (**Figure S13**). To search for functional signatures of different tissues, we performed GO enrichment analysis to characterize DEGs from the comparisons between different tissues. We found that upregulated genes in leaves were enriched in photosynthesis and monosaccharide catabolic processes. However, upregulated genes in roots were enriched in response to abiotic and biotic stimuli; upregulated genes in the rind were enriched in pathways related to transport, such as intercellular and carbohydrate transport. Compared with leaves and roots, upregulated genes in the pith were involved in carbohydrate transport and organic substance metabolic and biosynthetic processes. Additionally, upregulated genes in the pith relative to those in the rind were enriched in terms associated with fructose export from the vacuole to the cytoplasm, regulation of the syringal lignin biosynthetic process, plant-type cell wall organization or biogenesis. These results confirm that DEGs from different tissues are involved in biological pathways related to tissue-specific physiological functions.

### Identification of differentially methylated regions among different tissues

**Figure 5:**
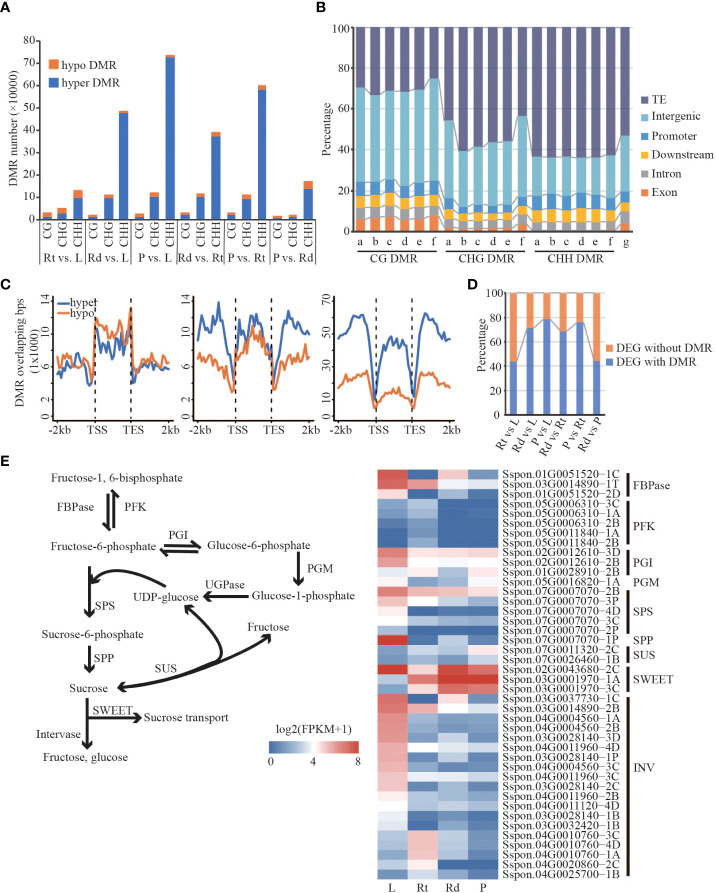
Differentially expressed genes are associated with differential methylation. **(A)** Barplot of hyper/hypo DMR. **(B)** The distribution of DMR in different regions of the genome. a, Rt vs. L; b, Rd vs. L; c, P vs. L; d, Rd vs. Rt; e, P vs. L; f, P vs. Rd; g, genome. **(C)** Distribution of DMR in the gene body and flanking region (Rt vs. L). **(D)** The proportion of DEG with DMR/without DMR. **(E)** Sucrose synthesis and hydrolysis pathways and the expression pattern of DMR-DEGs related to sucrose synthesis and hydrolysis pathways. FBPase, fructose-1,6-bisphosphatase; PFK, phosphofructokinase; PGI, phosphoglucose; PGM, phosphoglucomutase; SPS, sucrose phosphate synthase; SPP, sucrose-6F-phosphate phosphohydrolase; SUS, sucrose synthase; SWEET, sugars will eventually be exported transporters; INV, invertase; L, leaf; Rt, root; Rd, rind; P, pith; DEG, differentially expressed gene; DMR, differentially methylated region.

To characterize methylation changes among different tissues in sugarcane, we defined DMRs in each sequence context according to the method of Akalin (Akalin et al., 2012). A total of 113,536 CG-DMRs, 396,224 CHG-DMRs, and 1,146,516 CHH-DMRs were identified. Compared with CG and CHG DMRs, CHH DMRs were the most abundant across different comparisons among the four tissues. Meanwhile, compared with hypo-DMRs (lower DNA methylation in the right comparison), hyper-DMRs (higher methylation in the left comparison) were dominant (**Figure 5A**), consistent with the increased DNA methylation levels from leaf to root and then to rind and pith in the above analysis. Next, we examined the distribution of DMRs in different genomic features such as TE, intergenic, upstream, downstream, intron, and exon regions. As shown in **Figure 5B**, TE, intergenic, and genetic (upstream, downstream, intron, and exon) regions account for 53.52%, 27.45%, and 19.31% of the sugarcane genome, respectively. We found that CG DMRs are mainly located in the intergenic regions; Non-CG DMRs are mainly enriched in the TE regions, especially CHH methylation. This may indicate that CHH methylation changes mainly occur in the TE and intergenic regions (**Figure 5B**). Moreover, we found that many DMRs (~20%) were located in genic regions, including upstream, exon, intron, and downstream regions. Therefore, we hypothesized that DMRs adjacent to the gene regions might affect gene expression.

### Differential expression genes are associated with differentially methylated regions

We found substantial differences in gene expression and DNA methylation levels across different sugarcane tissues. In particular, many DMRs occur in the gene body and/or proximal regions, and these DMRs might contribute to changes in the expression of adjacent genes. From the above analysis, we found a large number of DMRs, including hyper- and hypo-methylated DMRs, in the gene body and flanking regions. Except for CG-DMRs, both CHG and CHH DMRs showed distinct distributions of hyper- and hypo-DMRs across the gene regions (**Figures 5C** and **Figure S14**). Strikingly, we observed that DMR-overlapped genes were more likely to be differentially expressed than DMR-non-overlapping genes, which was consistent across the comparisons between tissues (**Table S4**). These results indicate that changes in DNA methylation are associated with DEGs.

More than 40% of the DEGs contained DMRs across all six comparisons of the four tissues (**Figure 5D**). To understand how DMR-associated genes were associated with tissue divergence, we performed GO enrichment analysis of DMR-associated up- and down-regulated DEGs. Compared with the other three tissues, DMR-associated highly expressed genes in the roots were mainly involved in response to stress and root morphogenesis (**Figure S15**). For example, DMR-associated genes encoding phosphoinositide-specific phospholipase C (PI-PLC, *Sspon.05G0021570-2P*), class III peroxidase (PRX, *Sspon.01G0012950-1A*), and MYB (*Sspon.01G0019490-1A*) were highly expressed in roots, and their homologous genes in Arabidopsis were involved in growth, response to stresses, and lignin synthesis (Meijer and Munnik, 2003; Shigeto and Tsutsumi, 2016; Chezem et al., 2017). We also found that highly expressed DMR-associated DEGs in the leaves were significantly enriched in photosynthesis and sucrose-related pathways (**Figure S16**), such as photosynthesis, pigment metabolic process, and sucrose biosynthetic process. Furthermore, many biological processes related to sugar biosynthesis and metabolism were enriched in the DMR-associated DEGs (**Figure S16**). For example, *Sspon.02G0012860-2B* (**Figure S16**), which encodes NAD oxidoreductase, was upregulated in leaves. A recent study showed that NAD oxidoreductase was functional downstream of the photosynthetic electron transport chain and participated in the Calvin cycle, pigment synthesis (Pierella Karlusich and Carrillo, 2017), and is a key enzyme linking the light reaction of photosynthesis to carbon metabolism. Gene encoding inorganic pyrophosphatase (PPi, *Sspon.04G0005360-3D*) was highly expressed in leaves and its homologous genes in Arabidopsis are key enzymes in sucrose synthesis (Farré et al., 2000). Unlike leaves and roots, upregulated DMR-associated genes in the stem (rind and pith) were enriched in transport-related pathways such as sucrose and intracellular transport, cellular metabolic process, and hydrocarbon metabolic process. For example, *Sspon.04G0012730-4D* (Sugars Will Eventually be Exported Transporters; SWEET), *Sspon.01G005290-1A* (polyol/monosaccharide transporter 5), and *Sspon.01G0026170-1A* (Mfs transporter) encoding sugar transporters (**Figures S15** and **Figure S16**) were upregulated DMR-associated genes in the rinds. Sugar transporters function in sugar transport, distribution, and utilization in the phloem, as well as maintaining the balance between source and sink (Julius et al., 2017). *Sspon.02G0017170-1A* and *Sspon.02G0017170-3D* (**Figure S15**) were highly expressed DMR-associated genes in the piths encoding ADP-glucose pyrophosphorylase (AGPase); their homologous genes in Arabidopsis catalyze ADP glucose synthesis and release pyrophosphate, and are the key enzymes determining starch synthesis (Tetlow et al., 2004). *Sspon.02G0019390-3C* (**Figure S15**), encoding phosphoglucomutase, was upregulated in the DMR-associated genes in piths. In Arabidopsis, its homologous gene catalyzes the mutual conversion of glucose-1-phosphate and glucose-6-phosphate, key steps in sucrose metabolism and synthesis (Streb et al., 2009). We found that genes with many DMRs were highly expressed in the stem. In conclusion, DMR-associated DEGs in different sugarcane tissues are involved in essential biological pathways and have tissue-specific physiological functions that are closely related to photosynthesis, sugar metabolism, growth, and sugarcane development.

High sucrose accumulation is a characteristic feature of sugarcane. We found that DMR-associated DEGs were enriched in essential biological pathways (**Figures S15**, **S16**), such as sucrose synthesis, carbohydrate metabolism, and stress response. To investigate how DMR-associated genes contribute to the regulation of sucrose accumulation, we focused on the sucrose synthesis and hydrolysis pathways (**Figure 5E**). We observed that genes involved in the sucrose synthesis pathway, including *FBPase*, *PGI*, *SPS*, and *SPP*, were highly expressed in the leaves. However, in contrast to the other three tissues, genes encoding sucrose synthase (SUS) showed lower expression in leaves. These results suggest that sucrose synthesis in leaves mainly depends on the SPS-mediated pathway, consistent with previous studies (Buczynski et al., 1993; Verma et al., 2011). Moreover, *SWEETs* involved in sucrose transport were highly expressed in the leaves and stems, suggesting that the remaining sucrose was transported into sink tissues for consumption and storage, except for consumption in the leaves. Interestingly, we found that all *invertases* (INVs) involved in sucrose hydrolysis (**Figure 5E**) had a lower expression in stem tissue (rind and pith) than that in leaf and root tissue, indicating that sucrose transported to the stem was mainly used for storage, confirming our suspicion. Taken together, efficient sucrose synthesis in leaves, intense sucrose transport from leaves to stem, and low INV activity in the stem might be responsible for the high sucrose accumulation in sugarcane, indicating that DNA methylation-regulated genes function in high sucrose accumulation in sugarcane.

### Transcription factor genes are enriched in sugarcane DNA methylation valleys

**Figure 6:**
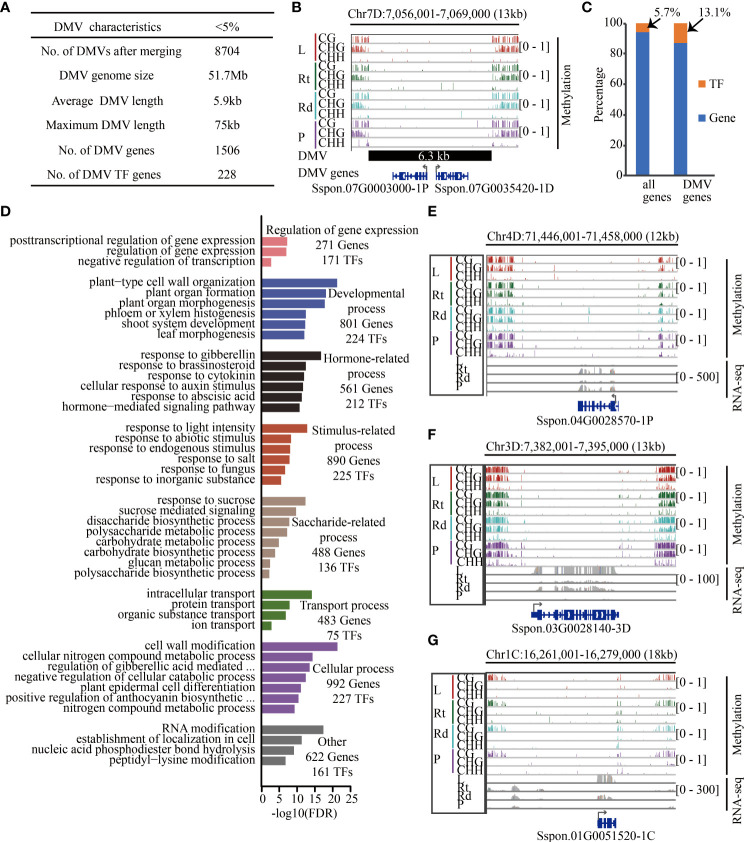
Transcription factors are enriched in sugarcane DMVs. **(A)** Summary of sugarcane DMV characteristics. **(B)** IGV of an 18-kb DMV located on chromosome 7D. Genes in blue color (Sspon.07G0003000-1P and Sspon.07G0035420-1D encoding the pectin lyase-like superfamily are involved in carbohydrate metabolic process) are located within this DMV, including 1 kb of 5′ and 3′ flanking regions. **(C)** Proportion of TF in genome and DMV regions. **(D)** Enriched GO terms with an FDR < 0.05. **(E–G)** Methylome and RNA-Seq genome browser views of three genes between at least two tissues. Red, green, cyan, and purple bars indicate leaf, root, rind, and pith, respectively. Gray collapsed bars indicate expression level. L, leaf; Rt, root; Rd, rind; P, pith; DMV, DNA methylation valley; TF, transcription factor; IGV, integrative genomics viewer.

Recent studies have shown that there are always lowly methylated or unmethylated regions in the genome, also known as DNA methylation valleys (DMVs) (Stadler et al., 2011; Lin et al., 2017; Chen et al., 2018; Li et al., 2018; Crisp et al., 2020). During soybean seed development, genes contained in DMVs tend to be enriched in tissue-specific biological pathways such as protein storage and fatty acid metabolism (Lin et al., 2017; Chen et al., 2018). Next, we scanned DNA methylome data from the four tissues for regions with <5% bulk methylation in all three cytosine sequence contexts as described in (Chen et al., 2018) and identified 28,531, 26,445, 25,311, and 24,666 DMVs in leaves, roots, rinds, and piths, respectively. Among these DMVs, 17,208 (2.9%), which were hypomethylated, were common to all four tissues and did not change significantly across different tissues. There were 8,704 non-redundant DMVs, accounting for 1.8% (51.7 Mb) of the genome length, which was significantly lower than the DMV ratio in other plants, implying species-specific DMV distribution (**Figure 6A**). For example, a 6.3-kb DMV exhibited low levels of all DNA methylation contexts across the four tissues and contained two protein-coding genes (**Figure 6B**).

We further examined the DMV regions and identified DMV genes if the gene body or flanking 1-kb regions overlapped with the DMV. We identified 1,734 genes located in DMVs, and transcription factors (TFs) (13.1%) were significantly enriched in these DMV genes (*p* < 2.2e-16, chi-squared test) (**Figure 6C**). GO enrichment analysis showed that these DMV genes were involved in regulating gene expression, developmental, stimulus-related, and saccharide-related processes (**Figure 6D**). In addition, we found that many TFs played important roles in these processes. For example, 488 DMV genes were associated with sucrose metabolism, of which 136 (28%) were TF encoding genes. Notably, many of these DMV TF genes were significantly differentially expressed in the four tissues (**Figure S17**). For example, the *bZIP* TF gene, *Sspon.04G0028570-1P*, was highly expressed in root tissue relative to the other tissues, and its homologous genes play an important role in biotic and abiotic stress in Arabidopsis (Droge-Laser et al., 2018) (**Figure 6E**). Moreover, *Sspon.06G0007540-2C* (**Figure S17**) encoding bZIP2 was higher in the piths than in the other three tissues, and the co-expression of its homologous genes *AtbZIP2* and *KIN10* in Arabidopsis activates *DIN6-LUC* to inhibit respiration (Baena-Gonzalez et al., 2007), suggesting that *Sspon.06G0007540-2C* might inhibit cellular respiration in the piths, reducing the consumption of sugar and facilitating sugar accumulation in the piths. In addition, some genes (non-TFs) located in the DMV regions were involved in the saccharide pathway. For example, *Sspon.03G0028140-3D* (**Figure 6F**) encoding SPS, a key gene regulating the conversion of photosynthetic products into sucrose and starch, was highly expressed in leaves (Verma et al., 2011). Furthermore, *Sspon.01G0051520-1C* (**Figure 6G**), which encodes FBPase involved in sucrose synthesis, was highly expressed in leaves—decreased *FBPase* expression inhibits sucrose synthesis (Strand et al., 2000; Lee et al., 2008). Therefore, *Sspon.03G0028140-3D* (**Figure 6F**) and *Sspon.01G0051520-1C* (**Figure 6G**) were highly expressed in leaves, suggesting that they played a role in transforming photosynthetic products and sucrose synthesis. Taken together, these data show that TFs and genes located in DMVs play essential roles in sugarcane development, stress response, and sucrose synthesis.

## Discussion

Publication of the sugarcane genome provided us with an unprecedented opportunity to investigate the role of DNA methylation in sugarcane. In the present study, we analyzed the dynamics of DNA methylation among tissues in sugarcane and the relationship between DNA methylation and gene expression, which will enhance knowledge in sugarcane epigenetics. DNA methylation levels are dynamically regulated by DNA methylases and demethylases (Law and Jacobsen, 2010; Zhang et al., 2018a). We observed that DNA methylation levels differed among the tissues (**Figure 1**). Furthermore, as shown in **Figure 3**, the expression patterns of *DME* and *ROS* in tissues are consistent with those of *MET1b* and *CMT3*, and the expression pattern of *Sspon.04G0012050-1A* (*ROS*) is consistent with those of *CMT2* and *DRM2* (*Sspon.01G0000660-1A*). Based on the fact that DNA demethylases can eliminate the mC of all sequence contexts (Choi et al., 2002; Gong et al., 2002; Morales-Ruiz et al., 2006; Ortega-Galisteo et al., 2008), we suggested that the DNA demethylation pathway plays a critical role in changes in methylation levels across the four tissues.

Cumulative evidence has shown that methylation of the gene body and flanking regions is involved in regulating gene expression (Wang et al., 2015; Xu et al., 2018; Zhang et al., 2018a; Wang et al., 2019; Cai et al., 2021). In tea plant (*Camellia sinensis*) (Tong et al., 2021), the methylation levels in all three sequence contexts of unexpressed genes were higher than those of expressed genes, the methylation levels of CHH in flanking regions of unexpressed genes were lower. However, CHH methylation patterns in sugarcane gene body and upstream regions were consistent with tea plant, whereas CG methylation pattern in gene body was opposite to that in the tea plant (Tong et al., 2021); the difference in CG methylation patterns in gene body between sugarcane and tea plant may be related to species specificity, such as genome size, and TE content. Moreover, CG methylation of gene body regions is always positively correlated with gene expression (Wang et al., 2015; Xu et al., 2018; Wang et al., 2019; Cai et al., 2021), but we observed that the highest-expressed genes did not have the highest CG methylation levels in the gene body (**Figures 4** and **Figure S11**). Methylation of the gene body can quantitatively impede transcript elongation in Arabidopsis (Zilberman et al., 2007). This may lead to the highest expression of genes without the highest CG methylation levels in the gene body. CG, CHG, and CHH methylation levels near the TSS were negatively correlated with gene expression (**Figures 4B** and **Figure S11**), similar to results for rice, soybean, apple (*Malus*), tea (*Camellia sinensis*), wild barley (*Hordeum vulgare*), Arabidopsis, and human (Zilberman et al., 2007; Laurent et al., 2010; Li et al., 2012; Song et al., 2013; Xu et al., 2018; Wang et al., 2019; Cai et al., 2021), demonstrating that methylation near the TSS is a common mechanism to suppress gene expression in eukaryotes. Additionally, highly expressed genes were correlated with higher CHH methylation levels in the promoter region (200–2,000 bp) close to the TSS (**Figures 4B** and **Figure S11**); a similar observation was made in soybean, maize, apple, and wild barley (Gent et al., 2013; Song et al., 2013; Xu et al., 2018; Cai et al., 2021). Taken together, the relationship between DNA methylation and gene expression is conserved in most of the studied plant species.

Genes regulated by DNA methylation are involved in several important biological pathways (Wang et al., 2015; Cheng et al., 2018; Wang et al., 2018; Xu et al., 2018; Wang et al., 2019). For example, highly expressed genes affected by DNA methylation in cassava are involved in carbohydrate metabolism, including hexose and glucose metabolism (Wang et al., 2015). Furthermore, upregulated genes regulated by DNA methylation during strawberry (*Fragaria* × *ananassa*) ripening are involved in fruit ripening-related processes, such as cytokinin and abscisic acid biosynthesis (Cheng et al., 2018). We found that DMR-DEGs in sugarcane were significantly enriched in biological pathways of tissue-specific physiological functions. For example, DMR-associated DEGs with higher expression in roots were significantly enriched in stress response and root morphogenesis (**Figures S15**, **S16**). Genes upregulated in leaves regulated by DNA methylation were involved in photosynthesis, hydrocarbon biosynthesis, and metabolic processes (**Figure S16**). DMR-associated DEGs that were highly expressed in the stem (rind and pith) were significantly enriched in transport-related pathways and metabolism-related processes, such as sucrose transport and hydrocarbon metabolic process (**Figures S15**, **S16**). In conclusion, DMR-associated DEGs between different tissues are involved in the biological pathways of tissue-specific physiological functions, which are essential for plant growth and development.

We observed that DMR-associated DEGs were enriched in important biological pathways (**Figures S15**, **S16**), such as sucrose synthesis, carbohydrate metabolism, and stress response. The high sucrose accumulation in sugarcane has attracted our attention to sucrose synthesis and hydrolysis pathways. As shown in **Figure 5E**, sucrose in leaves is mainly derived from the SPS-mediated sucrose synthesis pathway, and genes involved in sucrose transport are more highly expressed in stems than in leaves and roots. Moreover, INV involved in sucrose hydrolysis showed lower expression in the stem. Sugarcane has a universal source-sink system; except for consumption during leaf growth, the sucrose synthesized in leaves is exported to sink tissues and used for consumption and storage (Buczynski et al., 1993; Verma et al., 2011; Julius et al., 2017). Previous studies have indicated that SPS activity is a biochemical marker of high sucrose content in sugarcane (Verma et al., 2011). Collectively, we suggest that efficient sucrose synthesis in the leaves, intense sucrose transport to the stem, and low INV activity in the stem may be responsible for the high sucrose accumulation in sugarcane, indicating that DNA methylation plays an important role in sucrose accumulation in sugarcane.

Recent studies have shown that lowly methylated and unmethylated regions contain functional regulatory elements (Stadler et al., 2011; Lin et al., 2017; Chen et al., 2018; Li et al., 2018; Crisp et al., 2020). For instance, genes located in the DMVs of human embryonic stem cells or vertebrates, such as *Foxa1*, *Wnt1*, *GATA*, and *SOX2* (Stadler et al., 2011; Xie et al., 2013; Li et al., 2018), are involved in development and TF activity. DMVs during seed formation are enriched in TFs and development-related genes such as *WOX*, *PLETHORA*, *PIN1*, and *YUCCA4* (Lin et al., 2017; Chen et al., 2018). We also found many DMVs in sugarcane, which always overlapped with TFs, development, and sucrose-related genes such as *WRKY*, *bZIP*, *WOX*, *SPS*, and *FBPase* (**Figures 6D–G** and **Figure S17**), which function in sugarcane growth, morphogenesis, stress response, and carbohydrate metabolism, indicating that DMVs are common and essential for growth and development. Furthermore, approximately 40% of the genes (670 genes) located in the DMVs were differentially expressed between at least two tissues. Recent studies have shown that genes located in DMVs are enriched in H3K27me3 and H3K4me3 (Xie et al., 2013; Chen et al., 2018). Therefore, we hypothesized that DEGs located in sugarcane DMVs might be regulated by histone modification and TF regulation.

## Data availability statement

The original contributions presented in the study are publicly available. This data can be found here: NCBI, PRJNA730638.

## Author contributions

HW and BC conceived the study and supervised all parts of the project. CZo and YX collected samples and performed sequencing. YX, HY performed DNA methylation. YX and CZh performed transcriptome analysis and comparative analysis. YX and HW wrote the manuscript.

## Funding

This work was supported by the National Natural Science Foundation of China (No. 32160142) and Sugarcane Research Foundation of Guangxi University (Grant No. 2022GZA002) to HW and BC is supported by grant from Department of Science and Technology of Guangxi Zhuang Autonomous Region (AD17129002). YX is supported by Innovation Project of Guangxi Graduate Education (YCBZ2021005).

## Conflict of interest

The authors declare that the research was conducted in the absence of any commercial or financial relationships that could be construed as a potential conflict of interest.

## Publisher’s note

All claims expressed in this article are solely those of the authors and do not necessarily represent those of their affiliated organizations, or those of the publisher, the editors and the reviewers. Any product that may be evaluated in this article, or claim that may be made by its manufacturer, is not guaranteed or endorsed by the publisher.

## References

[B1] Akalin A. Kormaksson M. Li S. Garrett-Bakelman F. E. Figueroa M. E. Melnick A.. (2012). methylKit: A comprehensive r package for the analysis of genome-wide DNA methylation profiles. Genome Biol. 13, R87. doi: 10.1186/gb-2012-13-10-r87 23034086 PMC3491415

[B2] Ausin I. Feng S. Yu C. Liu W. Kuo H. Y. Jacobsen E. L.. (2016). DNA Methylome of the 20-gigabase Norway spruce genome. Proc. Natl. Acad. Sci. U.S.A. 113, E8106–E8113. doi: 10.1073/pnas.1618019113 27911846 PMC5167160

[B3] Baena-Gonzalez E. Rolland F. Thevelein J. M. Sheen J. (2007). A central integrator of transcription networks in plant stress and energy signalling. Nature 448, 938–942. doi: 10.1038/nature06069 17671505

[B4] Bolger A. M. Lohse M. Usadel B. (2014). Trimmomatic: A flexible trimmer for illumina sequence data. Bioinformatics 30, 2114–2120. doi: 10.1093/bioinformatics/btu170 24695404 PMC4103590

[B5] Buczynski S. R. Thom M. Chourey P. Maretzki A. (1993). Tissue distribution and characterization of sucrose synthase isozymes in sugarcane. J. Plant Physiol. 142, 641–646. doi: 10.1016/S0176-1617(11)80895-3

[B6] Cai S. Shen Q. Huang Y. Han Z. Wu D. Chen Z. H.. (2021). Multi-omics analysis reveals the mechanism underlying the edaphic adaptation in wild barley at evolution slope (Tabigha). Adv. Sci. (Weinh) 8, e2101374. doi: 10.1002/advs.202101374 34390227 PMC8529432

[B7] Chang Y. N. Zhu C. Jiang J. Zhang H. Zhu J. K. Duan C. G. (2020). Epigenetic regulation in plant abiotic stress responses. J. Integr. Plant Biol. 62, 563–580. doi: 10.1111/jipb.12901 31872527

[B8] Chan S. W. Henderson I. R. Zhang X. Shah G. Chien J. S. Jacobsen S. E. (2006). RNAi, DRD1, and histone methylation actively target developmentally important non-CG DNA methylation in arabidopsis. PloS Genet. 2, e83. doi: 10.1371/journal.pgen.0020083 16741558 PMC1472700

[B9] Cheng J. Niu Q. Zhang B. Chen K. Yang R. Zhu J. K.. (2018). Downregulation of RdDM during strawberry fruit ripening. Genome Biol. 19, 212. doi: 10.1186/s13059-018-1587-x 30514401 PMC6280534

[B10] Chen M. Lin J.-Y. Hur J. Pelletier J. M. Baden R. Pellegrini M.. (2018). Seed genome hypomethylated regions are enriched in transcription factor genes. Proc. Natl. Acad. Sci. 115, E8315. doi: 10.1073/pnas.1811017115 30104383 PMC6126732

[B11] Chezem W. R. Memon A. Li F. S. Weng J. K. Clay N. K. (2017). SG2-type R2R3-MYB transcription factor MYB15 controls defense-induced lignification and basal immunity in arabidopsis. Plant Cell 29, 1907–1926. doi: 10.1105/tpc.16.00954 28733420 PMC5590497

[B12] Choi Y. Gehring M. Johnson L. Hannon M. Harada J. J. Goldberg R. B.. (2002). DEMETER, a DNA glycosylase domain protein, is required for endosperm gene imprinting and seed viability in arabidopsis. Cell 110, 33–42. doi: 10.1016/s0092-8674(02)00807-3 12150995

[B13] Cokus S. J. Feng S. Zhang X. Chen Z. Merriman B. Haudenschild C. D.. (2008). Shotgun bisulphite sequencing of the arabidopsis genome reveals DNA methylation patterning. Nature 452, 215–219. doi: 10.1038/nature06745 18278030 PMC2377394

[B14] Crisp P. A. Marand A. P. Noshay J. M. Zhou P. Lu Z. Schmitz R. J.. (2020). Stable unmethylated DNA demarcates expressed genes and their cis-regulatory space in plant genomes. Proc. Natl. Acad. Sci. U.S.A. 117, 23991–24000. doi: 10.1073/pnas.2010250117 32879011 PMC7519222

[B15] Cuerda-Gil D. Slotkin R. K. (2016). Non-canonical RNA-directed DNA methylation. Nat. Plants 2, 16163. doi: 10.1038/nplants.2016.163 27808230

[B16] Droge-Laser W. Snoek B. L. Snel B. Weiste C. (2018). The arabidopsis bZIP transcription factor family-an update. Curr. Opin. Plant Biol. 45, 36–49. doi: 10.1016/j.pbi.2018.05.001 29860175

[B17] Farré E. M. Geigenberger P. Willmitzer L. Trethewey R. N. (2000). A possible role for pyrophosphate in the coordination of cytosolic and plastidial carbon metabolism within the potato tuber. Plant Physiol. 123, 681–688. doi: 10.1104/pp.123.2.681 10859198 PMC59036

[B18] Gent J. I. Ellis N. A. Guo L. Harkess A. E. Yao Y. Zhang X.. (2013). CHH islands: *de novo* DNA methylation in near-gene chromatin regulation in maize. Genome Res. 23, 628–637. doi: 10.1101/gr.146985.112 23269663 PMC3613580

[B19] Gong Z. Morales-Ruiz T. Ariza R. R. Roldán-Arjona T. David L. Zhu J. K. (2002). ROS1, a repressor of transcriptional gene silencing in arabidopsis, encodes a DNA glycosylase/lyase. Cell 111, 803–814. doi: 10.1016/s0092-8674(02)01133-9 12526807

[B20] Gouil Q. Baulcombe D. C. (2016). DNA Methylation signatures of the plant chromomethyltransferases. PloS Genet. 12, e1006526. doi: 10.1371/journal.pgen.1006526 27997534 PMC5221884

[B21] Huang X. Zhang S. Li K. Thimmapuram J. Xie S. Wren J. (2018). ViewBS: A powerful toolkit for visualization of high-throughput bisulfite sequencing data. Bioinformatics 34, 708–709. doi: 10.1093/bioinformatics/btx633 29087450 PMC5860610

[B22] Huerta-Cepas J. Szklarczyk D. Heller D. Hernández-Plaza A. Forslund S. K. Cook H.. (2019). eggNOG 5.0: A hierarchical, functionally and phylogenetically annotated orthology resource based on 5090 organisms and 2502 viruses. Nucleic Acids Res. 47, D309–d314. doi: 10.1093/nar/gky1085 30418610 PMC6324079

[B23] Julius B. T. Leach K. A. Tran T. M. Mertz R. A. Braun D. M. (2017). Sugar transporters in plants: New insights and discoveries. Plant Cell Physiol. 58, 1442–1460. doi: 10.1093/pcp/pcx090 28922744

[B24] Kankel M. W. Ramsey D. E. Stokes T. L. Flowers S. K. Haag J. R. Jeddeloh J. A.. (2003). Arabidopsis MET1 cytosine methyltransferase mutants. Genetics 163, 1109–1122. doi: 10.1093/genetics/163.3.1109 12663548 PMC1462485

[B25] Kawashima T. Berger F. (2014). Epigenetic reprogramming in plant sexual reproduction. Nat. Rev. Genet. 15, 613–624. doi: 10.1038/nrg3685 25048170

[B26] Kim D. Langmead B. Salzberg S. L. (2015). HISAT: A fast spliced aligner with low memory requirements. Nat. Methods 12, 357–360. doi: 10.1038/nmeth.3317 25751142 PMC4655817

[B27] Klopfenstein D. V. Zhang L. Pedersen B. S. Ramírez F. Warwick Vesztrocy A. Naldi A.. (2018). GOATOOLS: A Python library for gene ontology analyses. Sci. Rep. 8, 10872. doi: 10.1038/s41598-018-28948-z 30022098 PMC6052049

[B28] Laurent L. Wong E. Li G. Huynh T. Tsirigos A. Ong C. T.. (2010). Dynamic changes in the human methylome during differentiation. Genome Res. 20, 320–331. doi: 10.1101/gr.101907.109 20133333 PMC2840979

[B29] Law J. A. Jacobsen S. E. (2010). Establishing, maintaining and modifying DNA methylation patterns in plants and animals. Nat. Rev. Genet. 11, 204–220. doi: 10.1038/nrg2719 20142834 PMC3034103

[B30] Lee S. K. Jeon J. S. Bornke F. Voll L. Cho J. I. Goh C. H.. (2008). Loss of cytosolic fructose-1,6-bisphosphatase limits photosynthetic sucrose synthesis and causes severe growth retardations in rice (Oryza sativa). Plant Cell Environ. 31, 1851–1863. doi: 10.1111/j.1365-3040.2008.01890.x 18811733

[B31] Lindroth A. M. Cao X. Jackson J. P. Zilberman D. Mccallum C. M. Henikoff S.. (2001). Requirement of CHROMOMETHYLASE3 for maintenance of CpXpG methylation. Science 292, 2077–2080. doi: 10.1126/science.1059745 11349138

[B32] Lin J. Y. Le B. H. Chen M. Henry K. F. Hur J. Hsieh T. F.. (2017). Similarity between soybean and arabidopsis seed methylomes and loss of non-CG methylation does not affect seed development. Proc. Natl. Acad. Sci. U.S.A. 114, E9730–e9739. doi: 10.1073/pnas.1716758114 29078418 PMC5692608

[B33] Li Z. Wang M. Lin K. Xie Y. Guo J. Ye L.. (2019). The bread wheat epigenomic map reveals distinct chromatin architectural and evolutionary features of functional genetic elements. Genome Biol. 20, 139. doi: 10.1186/s13059-019-1746-8 31307500 PMC6628505

[B34] Li Y. Zheng H. Wang Q. Zhou C. Wei L. Liu X.. (2018). Genome-wide analyses reveal a role of polycomb in promoting hypomethylation of DNA methylation valleys. Genome Biol. 19, 18. doi: 10.1186/s13059-018-1390-8 29422066 PMC5806489

[B35] Li X. Zhu J. Hu F. Ge S. Ye M. Xiang H.. (2012). Single-base resolution maps of cultivated and wild rice methylomes and regulatory roles of DNA methylation in plant gene expression. BMC Genomics 13, 300. doi: 10.1186/1471-2164-13-300 22747568 PMC3447678

[B36] Meijer H. J. Munnik T. (2003). Phospholipid-based signaling in plants. Annu. Rev. Plant Biol. 54, 265–306. doi: 10.1146/annurev.arplant.54.031902.134748 14502992

[B37] Morales-Ruiz T. Ortega-Galisteo A. P. Ponferrada-Marín M. I. Martínez-Macías M. I. Ariza R. R. Roldán-Arjona T. (2006). DEMETER and REPRESSOR OF SILENCING 1 encode 5-methylcytosine DNA glycosylases. Proc. Natl. Acad. Sci. U.S.A. 103, 6853–6858. doi: 10.1073/pnas.0601109103 16624880 PMC1458983

[B38] Niederhuth C. E. Bewick A. J. Ji L. Alabady M. S. Kim K. D. Li Q.. (2016). Widespread natural variation of DNA methylation within angiosperms. Genome Biol. 17, 194. doi: 10.1186/s13059-016-1059-0 27671052 PMC5037628

[B39] Niu S. Li J. Bo W. Yang W. Zuccolo A. Giacomello S.. (2022). The Chinese pine genome and methylome unveil key features of conifer evolution. Cell 185, 204–217.e14. doi: 10.1016/j.cell.2021.12.006 34965378

[B40] Ortega-Galisteo A. P. Morales-Ruiz T. Ariza R. R. Roldán-Arjona T. (2008). Arabidopsis DEMETER-LIKE proteins DML2 and DML3 are required for appropriate distribution of DNA methylation marks. Plant Mol. Biol. 67, 671–681. doi: 10.1007/s11103-008-9346-0 18493721

[B41] Pertea M. Pertea G. M. Antonescu C. M. Chang T. C. Mendell J. T. Salzberg S. L. (2015). StringTie enables improved reconstruction of a transcriptome from RNA-seq reads. Nat. Biotechnol. 33, 290–295. doi: 10.1038/nbt.3122 25690850 PMC4643835

[B42] Pierella Karlusich J. J. Carrillo N. (2017). Evolution of the acceptor side of photosystem I: ferredoxin, flavodoxin, and ferredoxin-NADP(+) oxidoreductase. Photosynth Res. 134, 235–250. doi: 10.1007/s11120-017-0338-2 28150152

[B43] Sahraeian S. M. E. Mohiyuddin M. Sebra R. Tilgner H. Afshar P. T. Au K. F.. (2017). Gaining comprehensive biological insight into the transcriptome by performing a broad-spectrum RNA-seq analysis. Nat. Commun. 8, 59–59. doi: 10.1038/s41467-017-00050-4 28680106 PMC5498581

[B44] Shigeto J. Tsutsumi Y. (2016). Diverse functions and reactions of class III peroxidases. New Phytol. 209, 1395–1402. doi: 10.1111/nph.13738 26542837

[B45] Song Q. Guan X. Chen Z. J. (2015). Dynamic roles for small RNAs and DNA methylation during ovule and fiber development in allotetraploid cotton. PloS Genet. 11, e1005724. doi: 10.1371/journal.pgen.1005724 26710171 PMC4692501

[B46] Song Q. X. Lu X. Li Q. T. Chen H. Hu X. Y. Ma B.. (2013). Genome-wide analysis of DNA methylation in soybean. Mol. Plant 6, 1961–1974. doi: 10.1093/mp/sst123 23966636

[B47] Stadler M. B. Murr R. Burger L. Ivanek R. Lienert F. Schöler A.. (2011). DNA-Binding factors shape the mouse methylome at distal regulatory regions. Nature 480, 490–495. doi: 10.1038/nature10716 22170606

[B48] Strand A. Zrenner R. Trevanion S. Stitt M. Gustafsson P. Gardeström P. (2000). Decreased expression of two key enzymes in the sucrose biosynthesis pathway, cytosolic fructose-1,6-bisphosphatase and sucrose phosphate synthase, has remarkably different consequences for photosynthetic carbon metabolism in transgenic arabidopsis thaliana. Plant J. 23, 759–770. doi: 10.1046/j.1365-313x.2000.00847.x 10998187

[B49] Streb S. Egli B. Eicke S. Zeeman S. C. (2009). The debate on the pathway of starch synthesis: A closer look at low-starch mutants lacking plastidial phosphoglucomutase supports the chloroplast-localized pathway. Plant Physiol. 151, 1769–1772. doi: 10.1104/pp.109.144931 19776162 PMC2785970

[B50] Stroud H. Do T. Du J. Zhong X. Feng S. Johnson L.. (2014). Non-CG methylation patterns shape the epigenetic landscape in arabidopsis. Nat. Struct. Mol. Biol. 21, 64–72. doi: 10.1038/nsmb.2735 24336224 PMC4103798

[B51] Tetlow I. J. Morell M. K. Emes M. J. (2004). Recent developments in understanding the regulation of starch metabolism in higher plants. J. Exp. Bot. 55, 2131–2145. doi: 10.1093/jxb/erh248 15361536

[B52] Tong W. Li R. Huang J. Zhao H. Ge R. Wu Q.. (2021). Divergent DNA methylation contributes to duplicated gene evolution and chilling response in tea plants. Plant J. 106, 1312–1327. doi: 10.1111/tpj.15237 33730390

[B53] Turco G. M. Kajala K. Kunde-Ramamoorthy G. Ngan C. Y. Olson A. Deshphande S.. (2017). DNA Methylation and gene expression regulation associated with vascularization in sorghum bicolor. New Phytol. 214, 1213–1229. doi: 10.1111/nph.14448 28186631 PMC5655736

[B54] Verma A. K. Upadhyay S. K. Verma P. C. Solomon S. Singh S. B. (2011). Functional analysis of sucrose phosphate synthase (SPS) and sucrose synthase (SS) in sugarcane (Saccharum) cultivars. Plant Biol. (Stuttg) 13, 325–332. doi: 10.1111/j.1438-8677.2010.00379.x 21309979

[B55] Wang H. Beyene G. Zhai J. Feng S. Fahlgren N. Taylor N. J.. (2015). CG gene body DNA methylation changes and evolution of duplicated genes in cassava. Proc. Natl. Acad. Sci. U.S.A. 112, 13729–13734. doi: 10.1073/pnas.1519067112 26483493 PMC4640745

[B56] Wang W. S. Pan Y. J. Zhao X. Q. Dwivedi D. Zhu L. H. Ali J.. (2011). Drought-induced site-specific DNA methylation and its association with drought tolerance in rice (Oryza sativa l.). J. Exp. Bot. 62, 1951–1960. doi: 10.1093/jxb/erq391 21193578 PMC3060682

[B57] Wang L. Shi Y. Chang X. Jing S. Zhang Q. You C.. (2019). DNA Methylome analysis provides evidence that the expansion of the tea genome is linked to TE bursts. Plant Biotechnol. J. 17, 826–835. doi: 10.1111/pbi.13018 30256509 PMC6419580

[B58] Wang L. Xie J. Hu J. Lan B. You C. Li F.. (2018). Comparative epigenomics reveals evolution of duplicated genes in potato and tomato. Plant J. 93, 460–471. doi: 10.1111/tpj.13790 29178145

[B59] Wang Q. Xu J. Pu X. Lv H. Liu Y. Ma H.. (2021). Maize DNA methylation in response to drought stress is involved in target gene expression and alternative splicing. Int. J. Mol. Sci. 22, 8285–8303. doi: 10.3390/ijms22158285 34361051 PMC8347047

[B60] Xie W. Schultz M. D. Lister R. Hou Z. Rajagopal N. Ray P.. (2013). Epigenomic Analysis of Multilineage Differentiation of Human Embryonic Stem Cells. Cell 153, 1134–1148. doi: 10.1016/j.cell.2013.04.022 23664764 PMC3786220

[B61] Xi Y. Li W. (2009). BSMAP: whole genome bisulfite sequence MAPping program. BMC Bioinf. 10, 232. doi: 10.1186/1471-2105-10-232 PMC2724425 19635165

[B62] Xu J. Zhou S. Gong X. Song Y. Van Nocker S. Ma F.. (2018). Single-base methylome analysis reveals dynamic epigenomic differences associated with water deficit in apple. Plant Biotechnol. J. 16, 672–687. doi: 10.1111/pbi.12820 28796917 PMC5787839

[B63] Zemach A. Kim M. Y. Hsieh P. H. Coleman-Derr D. Eshed-Williams L. Thao K.. (2013). The arabidopsis nucleosome remodeler DDM1 allows DNA methyltransferases to access H1-containing heterochromatin. Cell 153, 193–205. doi: 10.1016/j.cell.2013.02.033 23540698 PMC4035305

[B64] Zhang H. Lang Z. Zhu J. K. (2018a). Dynamics and function of DNA methylation in plants. Nat. Rev. Mol. Cell Biol. 19, 489–506. doi: 10.1038/s41580-018-0016-z 29784956

[B65] Zhang J. Zhang X. Tang H. Zhang Q. Hua X. Ma X.. (2018b). Allele-defined genome of the autopolyploid sugarcane saccharum spontaneum l. Nat. Genet. 50, 1565–1573. doi: 10.1038/s41588-018-0237-2 30297971

[B66] Zhu N. Cheng S. Liu X. Du H. Dai M. Zhou D. X.. (2015). The R2R3-type MYB gene OsMYB91 has a function in coordinating plant growth and salt stress tolerance in rice. Plant Sci. 236, 146–156. doi: 10.1016/j.plantsci.2015.03.023 26025528

[B67] Zilberman D. Gehring M. Tran R. K. Ballinger T. Henikoff S. (2007). Genome-wide analysis of arabidopsis thaliana DNA methylation uncovers an interdependence between methylation and transcription. Nat. Genet. 39, 61–69. doi: 10.1038/ng1929 17128275

