# Supplementary material for: Dynamic DNA methylation changes reveal tissue-specific gene expression in sugarcane
Source: Front Plant Sci. 2022 Oct 13;13:1036764. doi: 10.3389/fpls.2022.1036764 (PMC9606695; doi:10.3389/fpls.2022.1036764)
Supplement: Supplementary file 1 [file DataSheet_1.pdf]

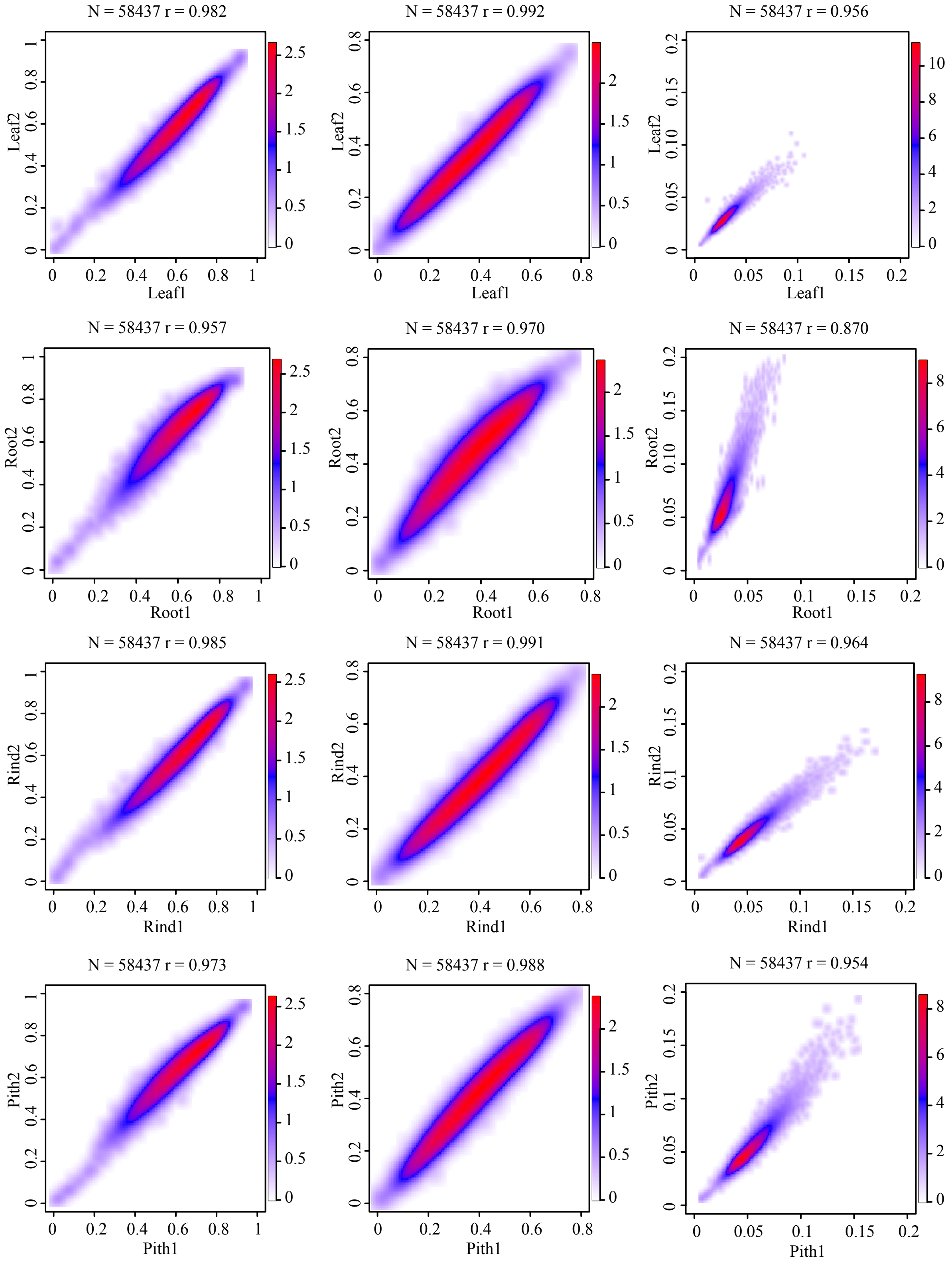

Supplementary Figure 1. The correlation of replication of BS-seq in sugarcane.

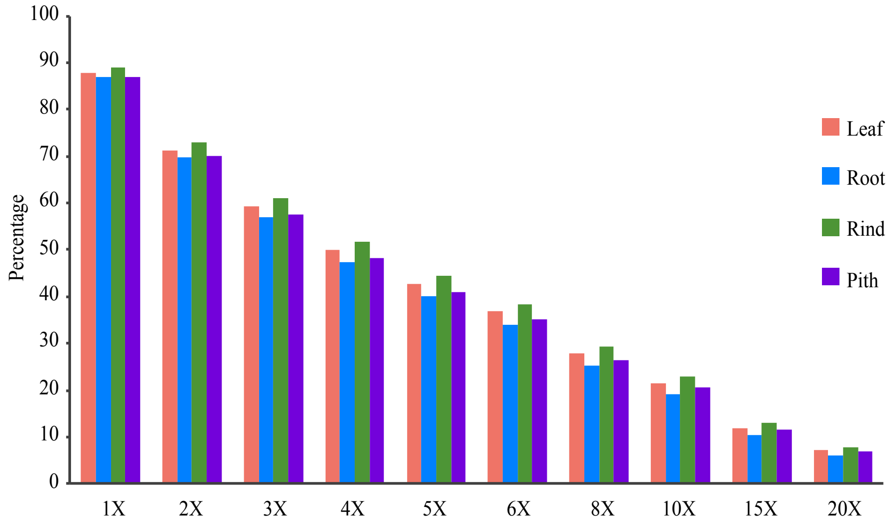

Supplementary Figure 2. BS-Seq coverage was shown as the proportion of cytosines that were covered by at least 'X' reads.

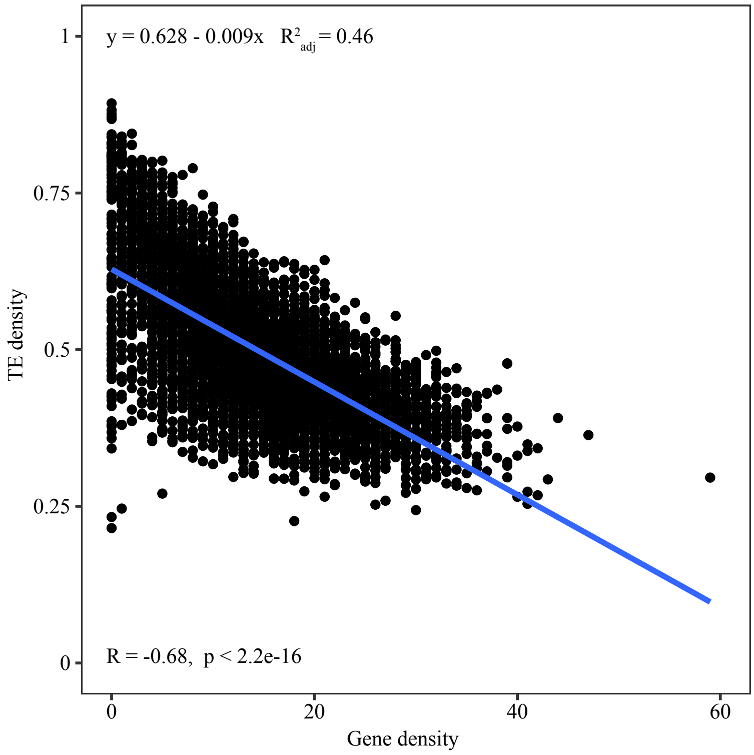

Supplementary Figure 3. The correlation between gene density and TE density. TE percentage is the ratio of TE length to 500kb windows .

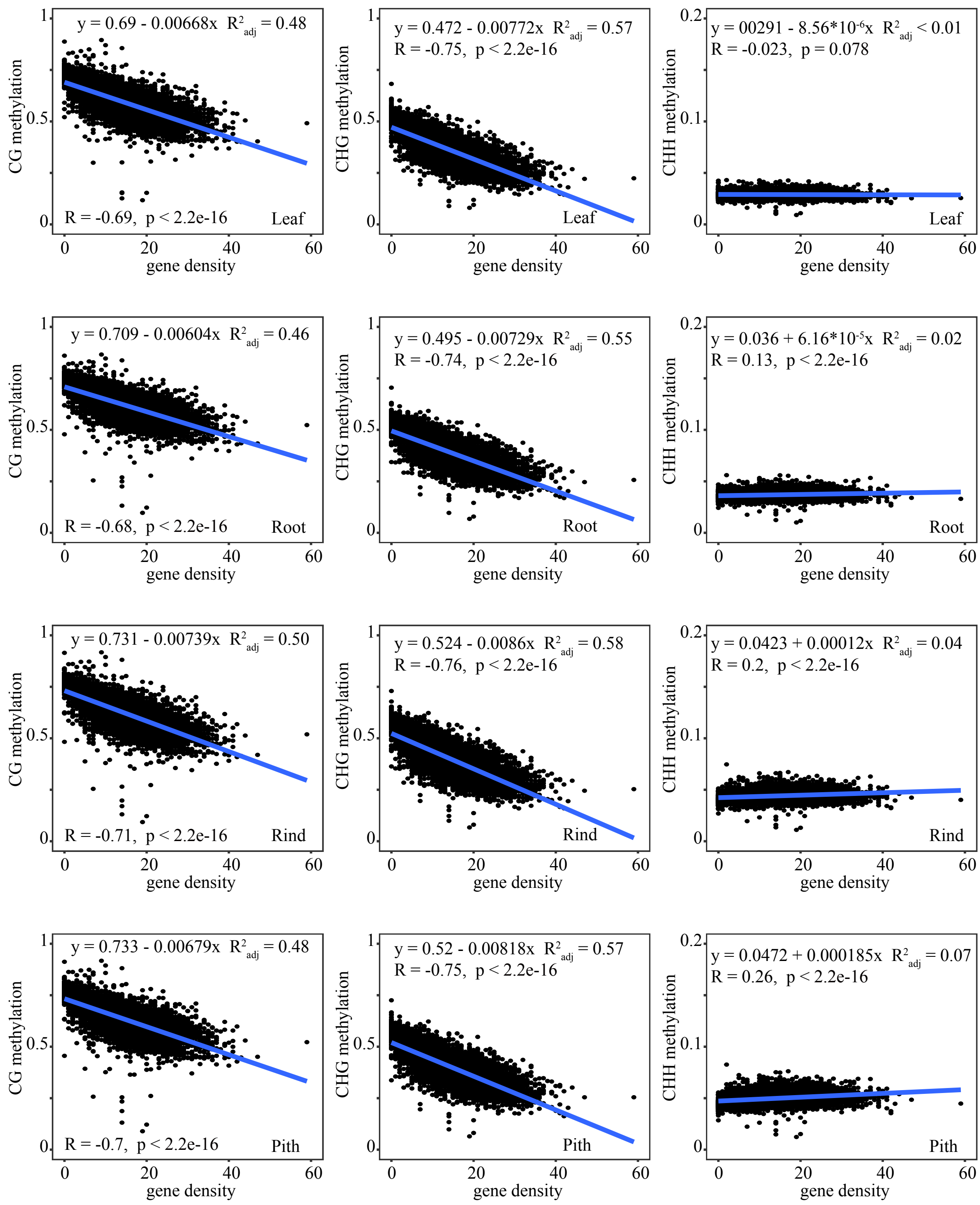

Supplementary Figure 4. The correlation between gene density and the methylation level in three contexts. The methylation level and gene density were counted in a slide window of 500 kb.

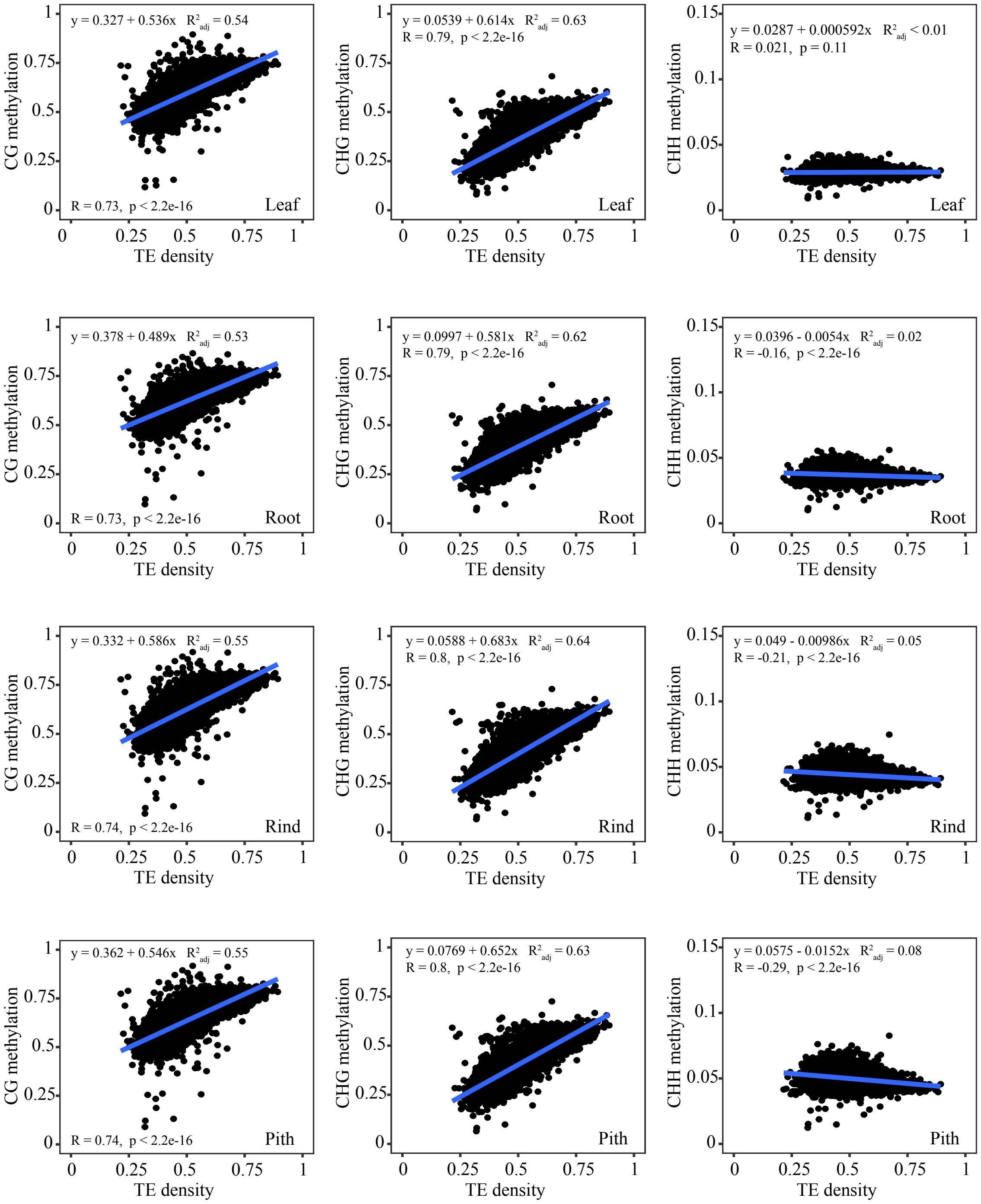

Supplementary Figure 5. The correlation between the TE density and the methylation level in three contexts. The methylation level and the TE density were counted in a slide window of 500 kb.

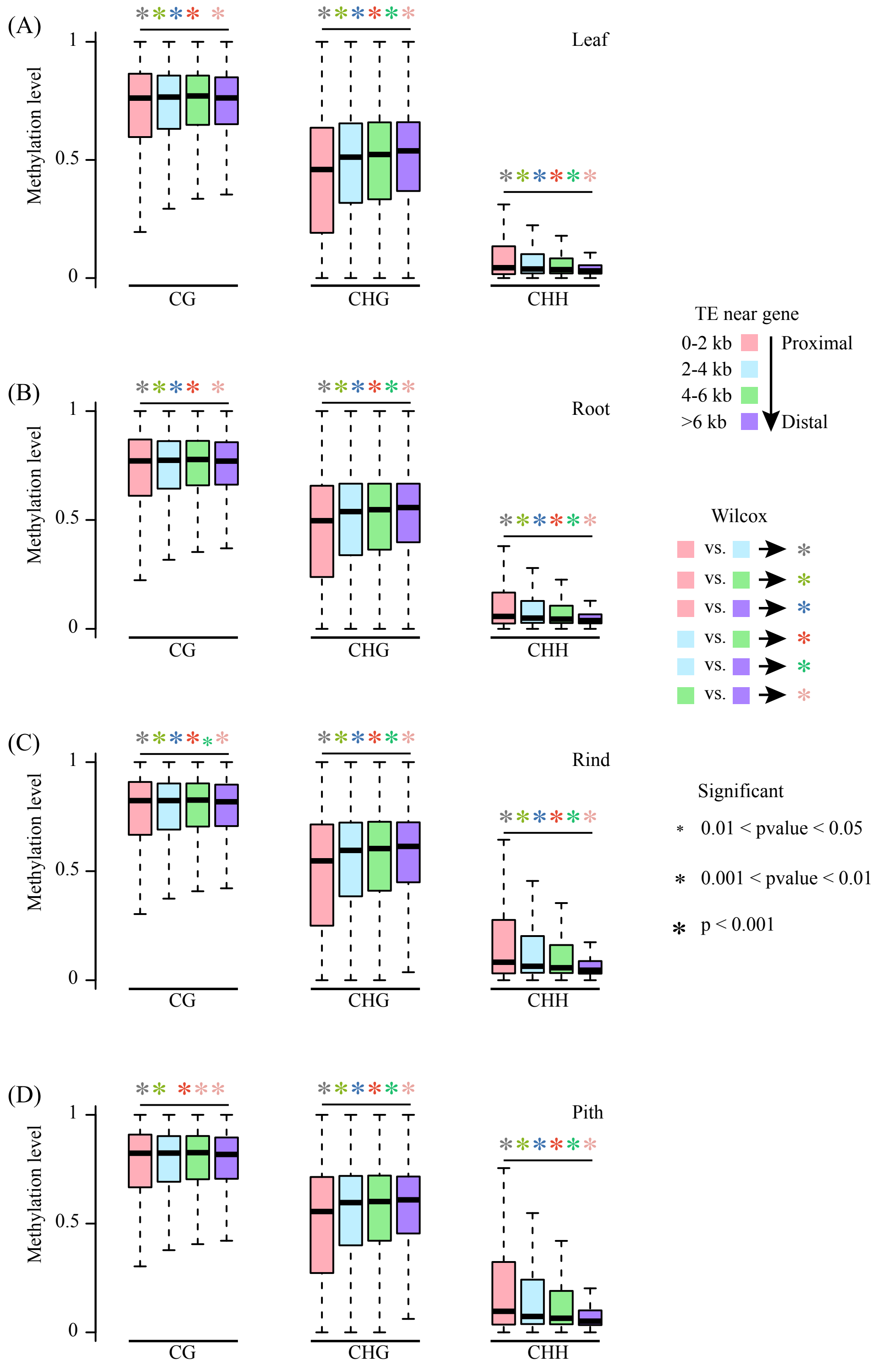

Supplementary Figure 6. Methylation levels in TEs relative to the distance from the nearest gene. **(A-D)** The methylation levels of TE in leaf, root, rind and pith, respectively. Methylation differences between different groups were tested using the Wilcoxon rank-sum test. The colors of the asterisk (*) represent the comparison between different clusters, and the size of the asterisk (*) indicates the criterion of significance.

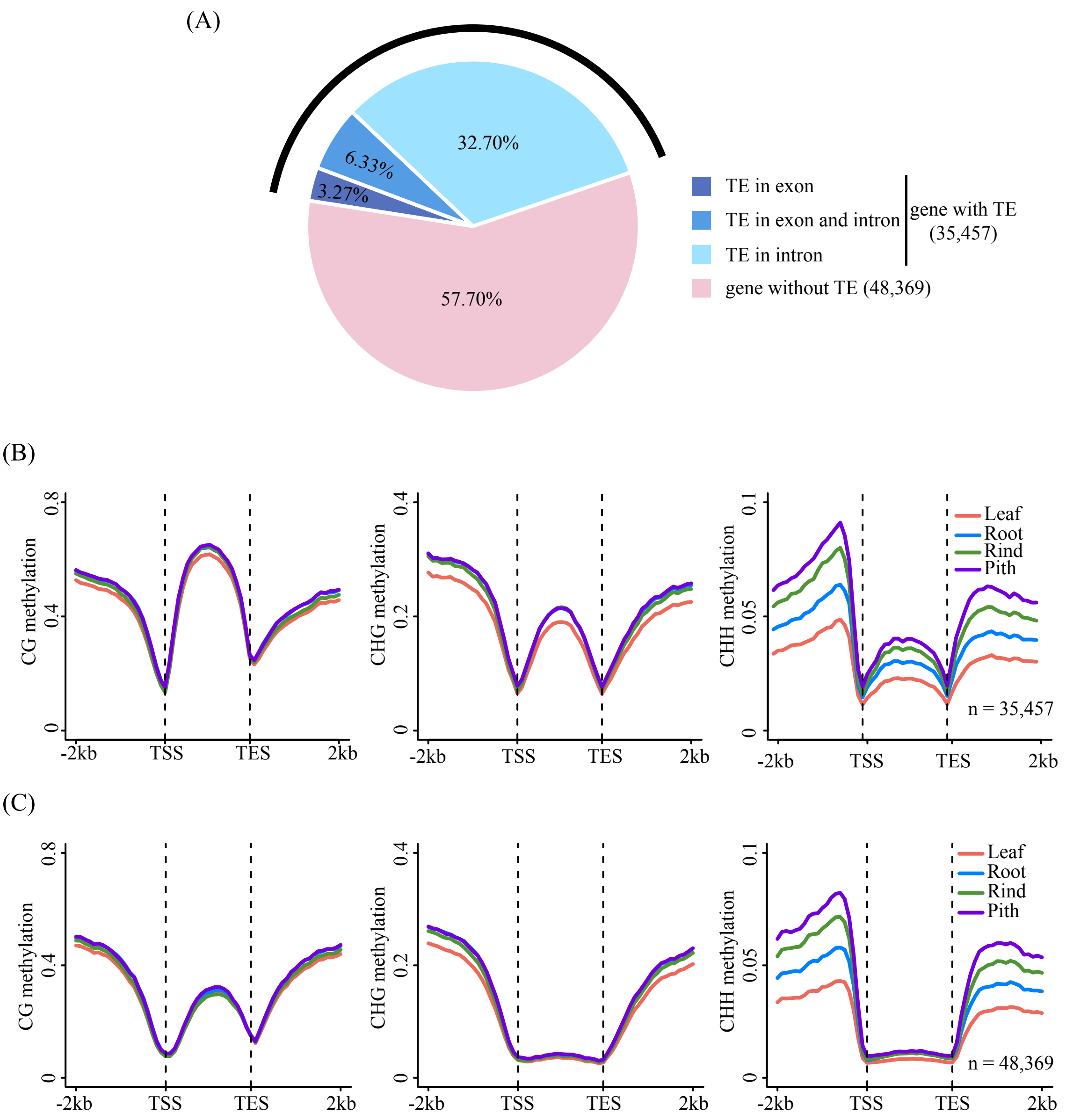

Supplementary Figure 7. DNA methylation patterns of protein-coding genes. **(A)** The percentage of gene with TE and without TE in genebody. **(B)** The metaplot of genes with TE in genebody. **(C)** The metaplot of genes without TE in genebody.

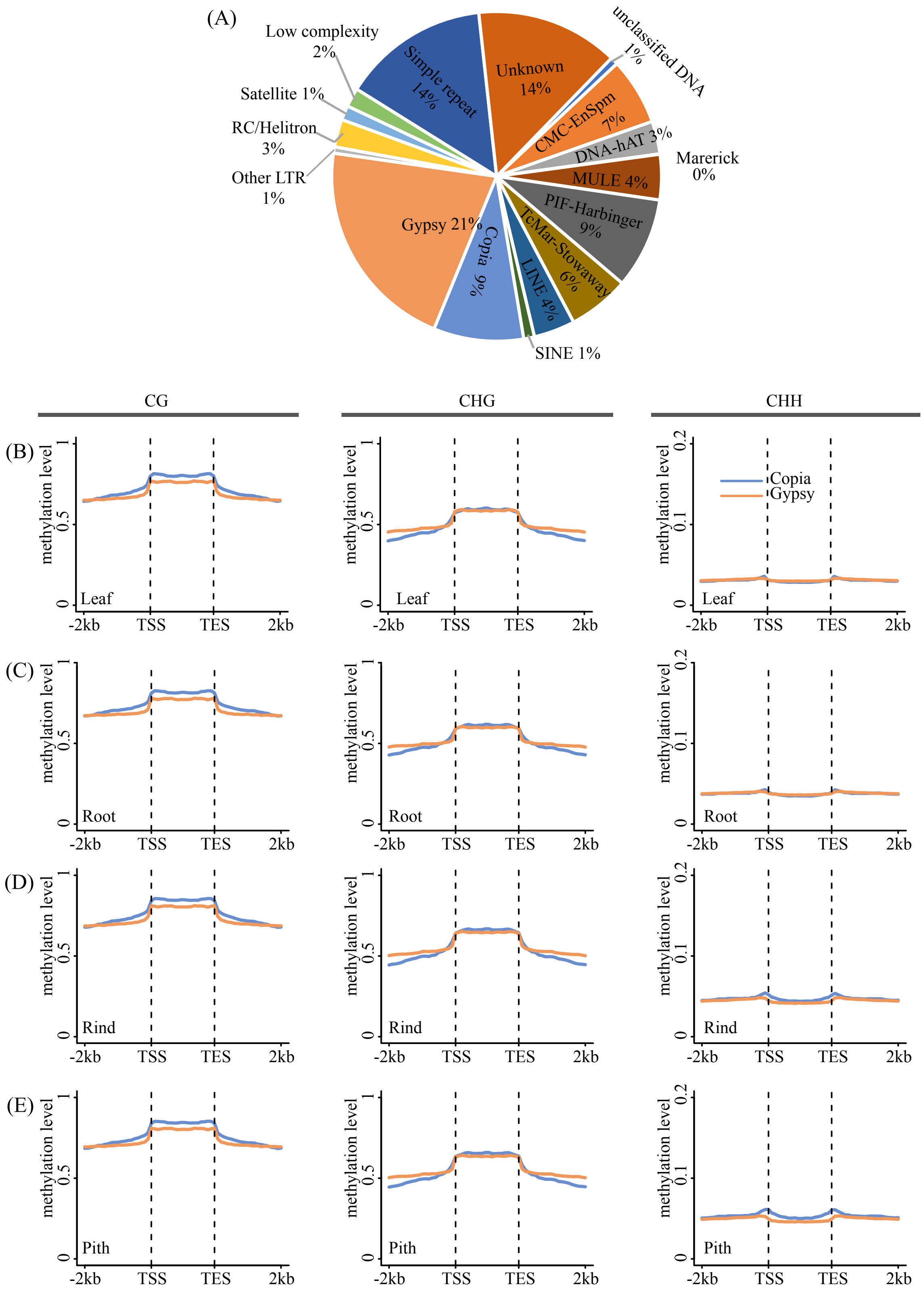

Supplementary Figure 8. TE in different families. **(A)** The percentage of different TE families. **(B-E)** The metaplot of Copia and Gypsy in leaf, root, rind and pith, respectively; CG (left), CHG (middle), CHH (right).

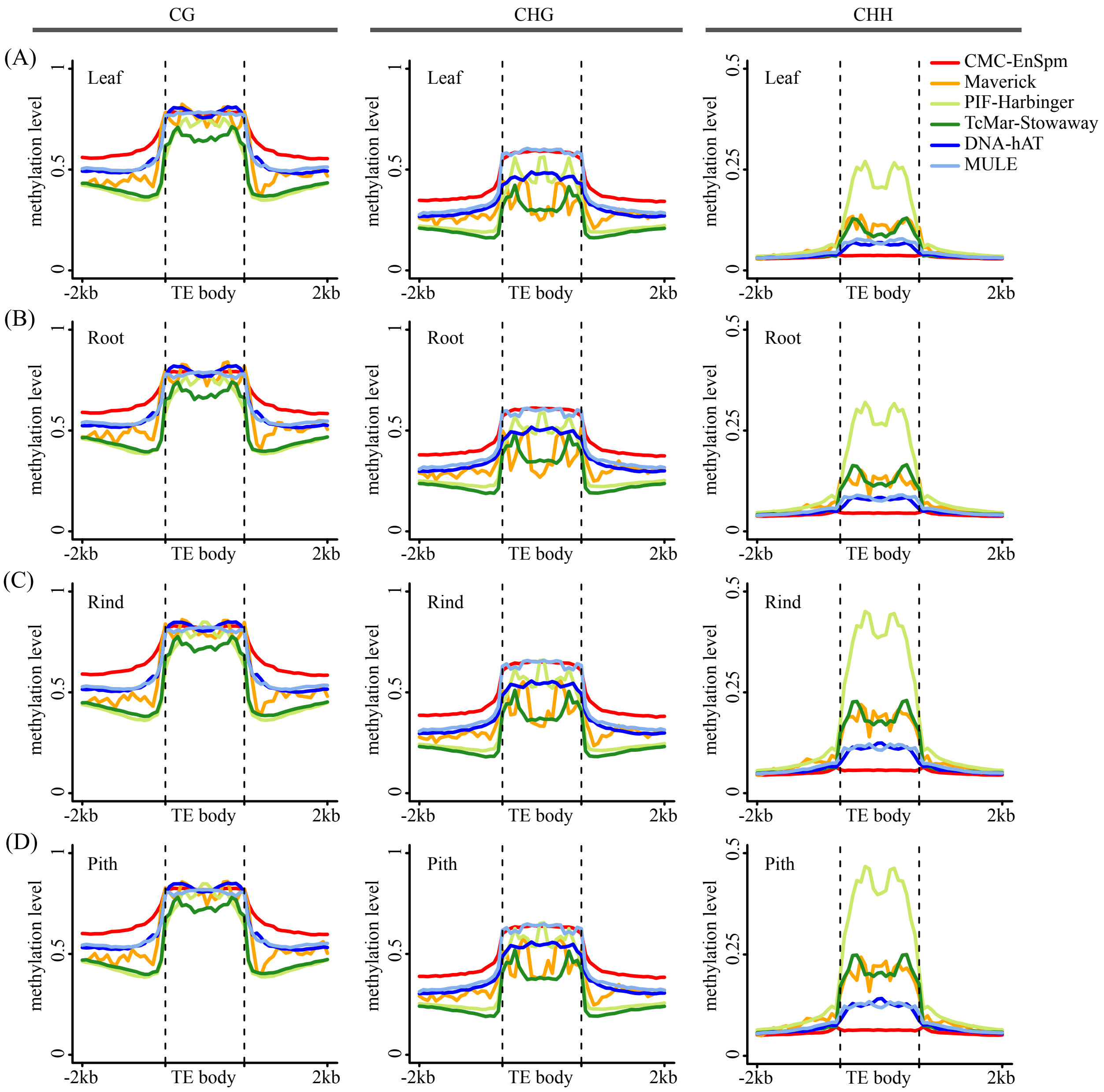

Supplementary Figure 9. DNA methylation patterns of Class II TE in different families. **(A-D)** The metaplot of TE families in leaf, root, rind and pith, respectively; CG (left), CHG (middle), CHH (right).

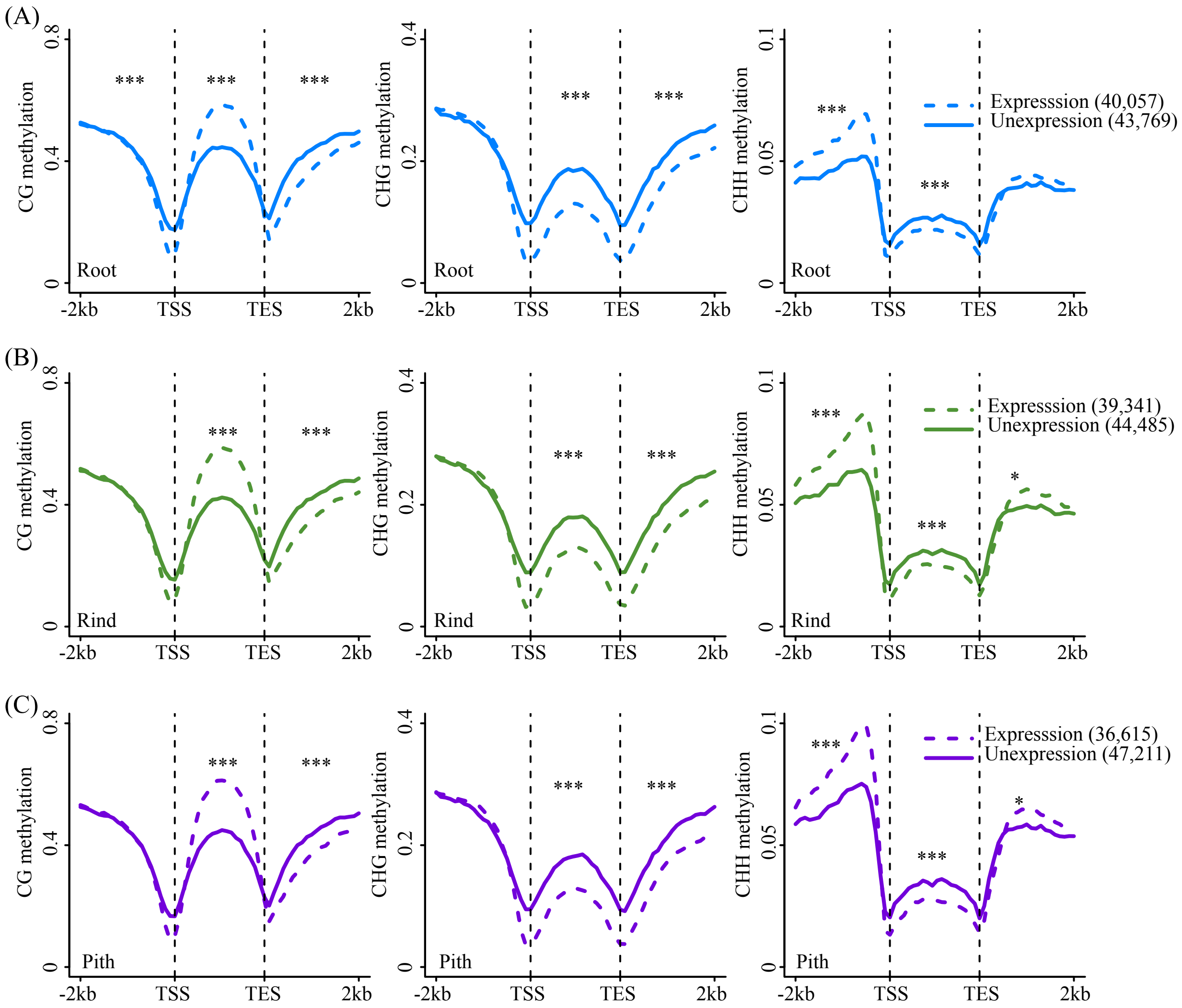

Supplementary Figure 10. Methylation level changes between the expressed and unexpressed genes in CG, CHG, and CHH sequence contexts. **(A-C)** The metaplots are root, rind and pith, respectively. expressed genes (FPKM $\geq$ 1), unexpressed genes (FPKM < 1). Methylation differences between expressed genes and unexpressed genes were tested using the Wilcoxon rank-sum test. * (0.01 < pvalue < 0.05), ** (0.001 < pvalue < 0.01), *** (pvalue < 0.001). CG (left), CHG (middle), CHH (right).

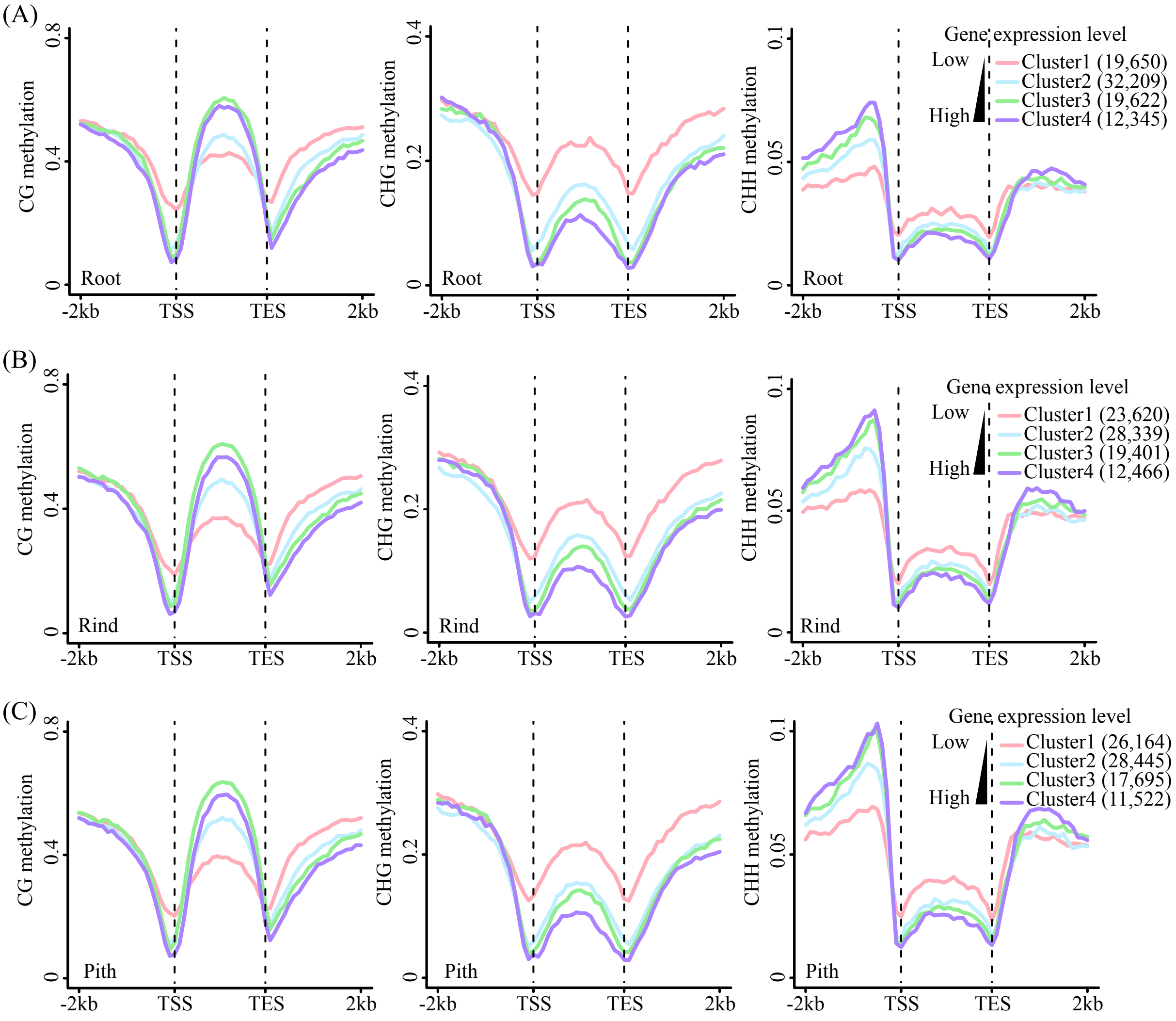

Supplementary Figure 11. Correlations between methylation levels (CG, CHG and CHH) and gene expression across gene body and flanking regions. **(A-C)** The metaplots are root, rind and pith, respectively. Methylation level of each gene group [Cluster1 (FPKM = 0), Cluster2 (0 < FPKM ≤ 2), Cluster3 (2 < FPKM ≤ 10), Cluster4 (FPKM ≥ 10)] were calculated. CHG (middle), CHH (right).

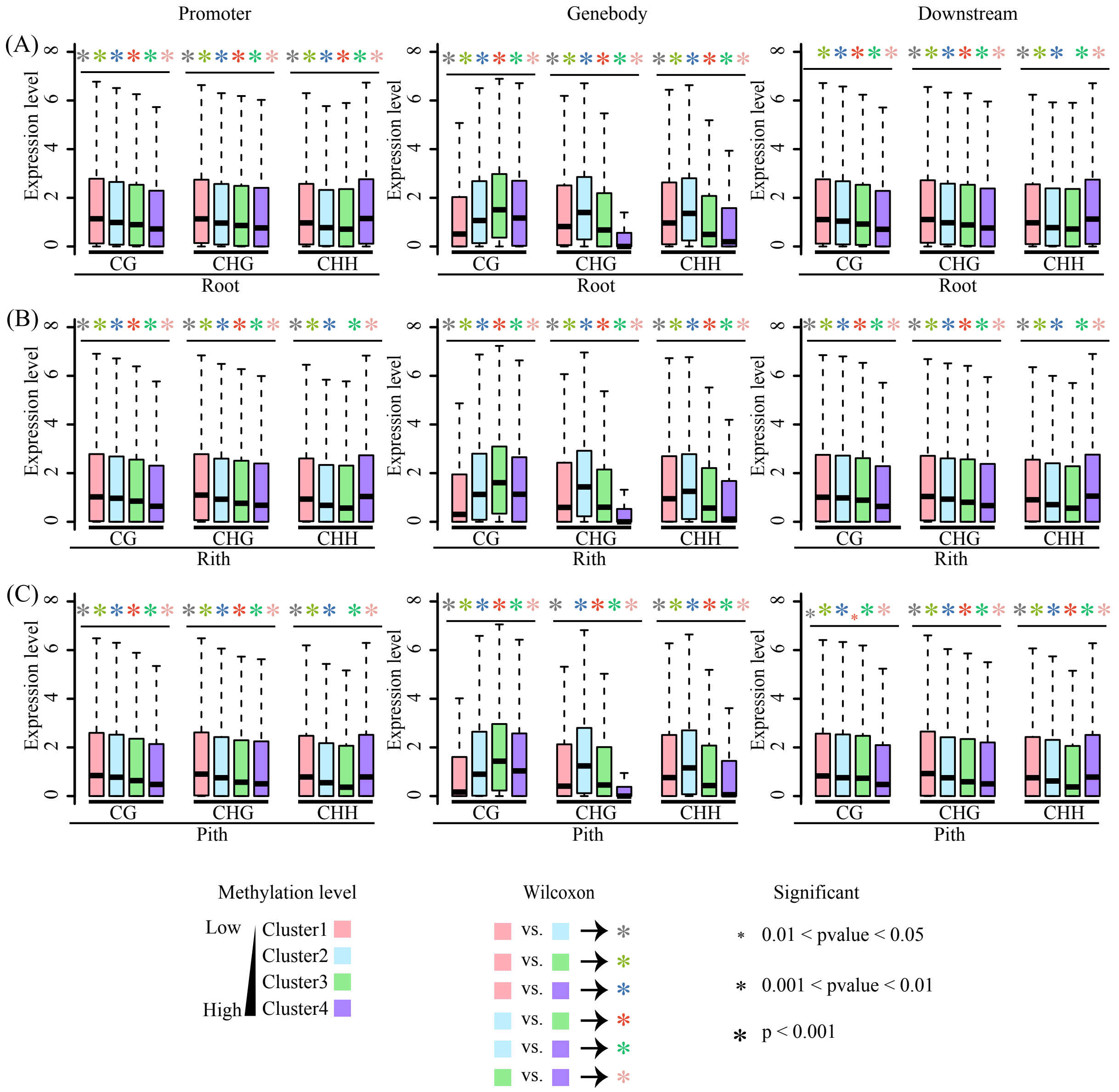

Supplementary Figure 12. Expression levels of methylated genes in genebody and flanking. **(A-C)** Expression levels of methylated genes in root, rind and pith, respectively. Genes were divided into four quartiles based on methylation levels, from the first quartile (the most lowly methylated 25% of genes) to the fourth quartile (the most highly methylated 25% of genes). Expression differences between differeent clusters were tested by using the Wilcoxon rank sum test, the colors of the asterisk (*) represent the comparison between different clusters, and the size of the asterisk (*) indicates the criterion of significance. Promoter (left), genebody (middle) and downstream (right)

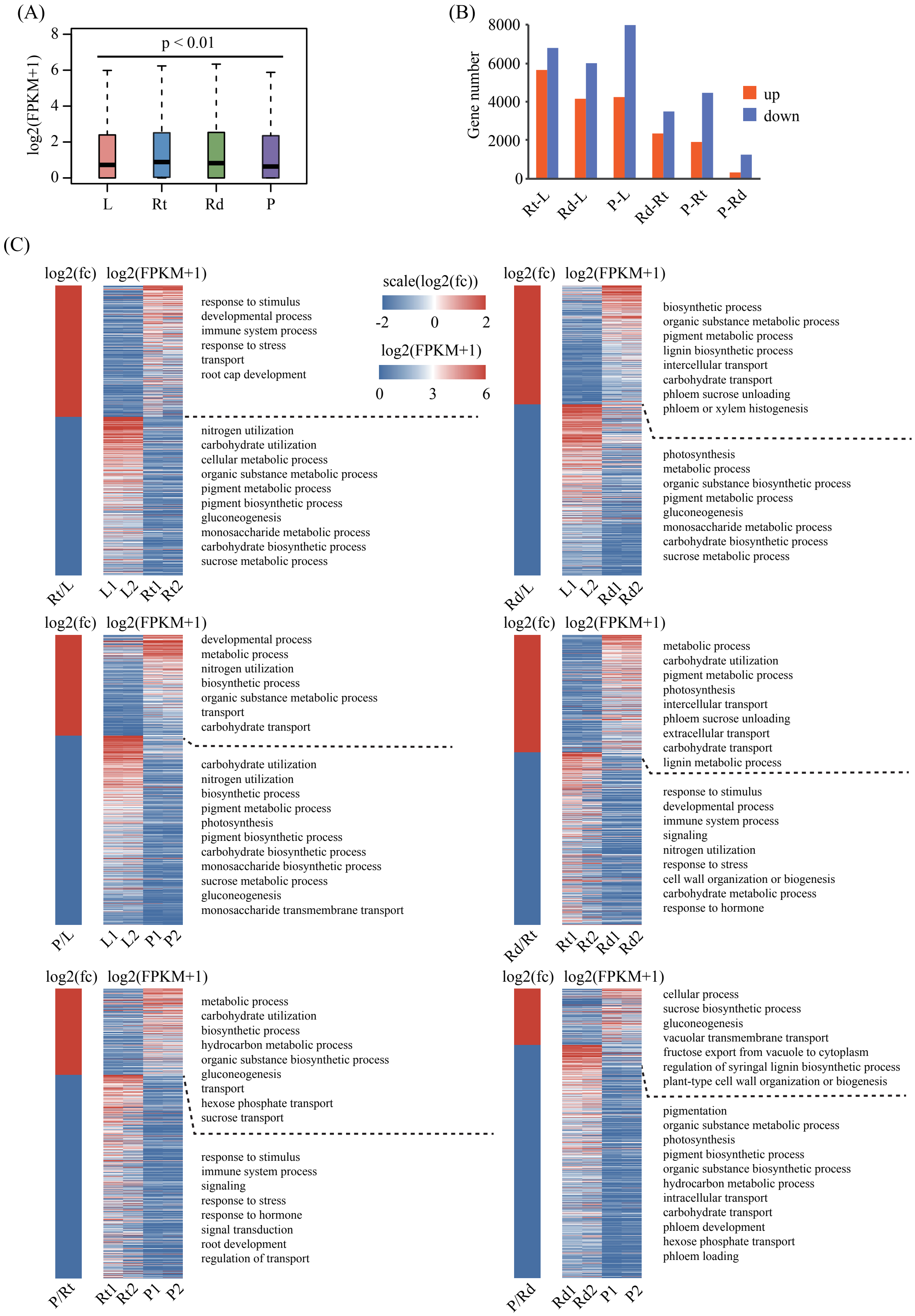

Supplementary Figure 13. Differential expression genes among different tissues in sugarcane. **(A)** Gene expression distribution among different tissues. Expression differences between differeent tissues were tested by using the Wilcoxon rank-sum test. **(B)** The distribution of DEGs among different tissues. **(C)** Heatmaps of the significantly up- or down-regulated transcripts genes among different tissues in sugarcane. L, leaf; Rt, root; Rd, rind; P, pith. fc, fold change; FPKM, fragments per kilobase of transcript per million fragments mapped.

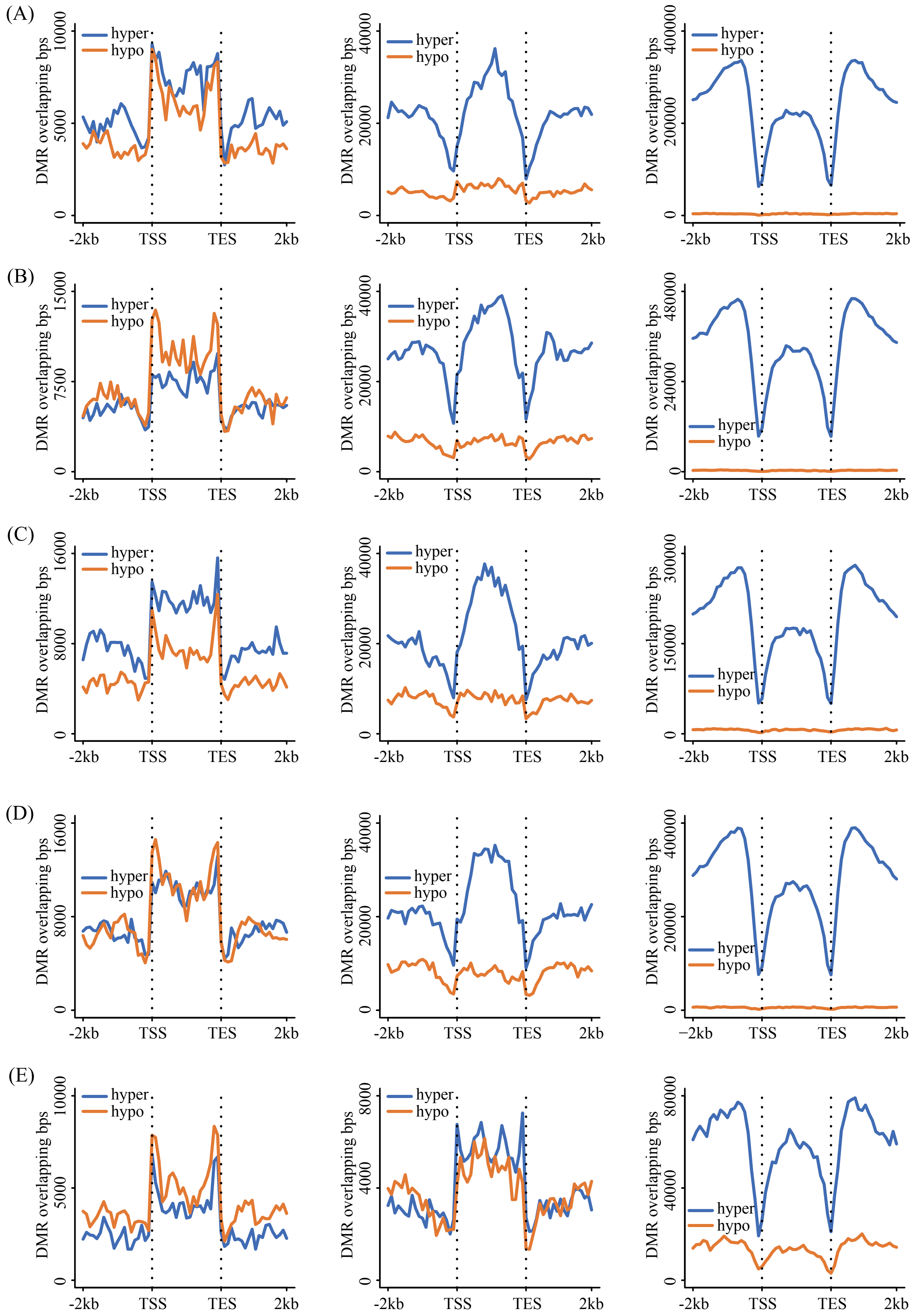

Supplementary Figure 14. Distribution of DMR sequences around gene and flangking. **(A)** The distribution of DMR (Rind vs. Leaf); **(B)** The distribution of DMR (Pith vs. Leaf); **(C)** The distribution of DMR (Rind vs. Root); **(D)** The distribution of DMR (Pith vs. Root); **(E)** The distribution of DMR (Pith vs. Rind). hypo-DMRs (lower DNA methylation in right tissue), hyper-DMRs (higher methylation in left tissue). CG (left), CHG (middle), CHH (right).

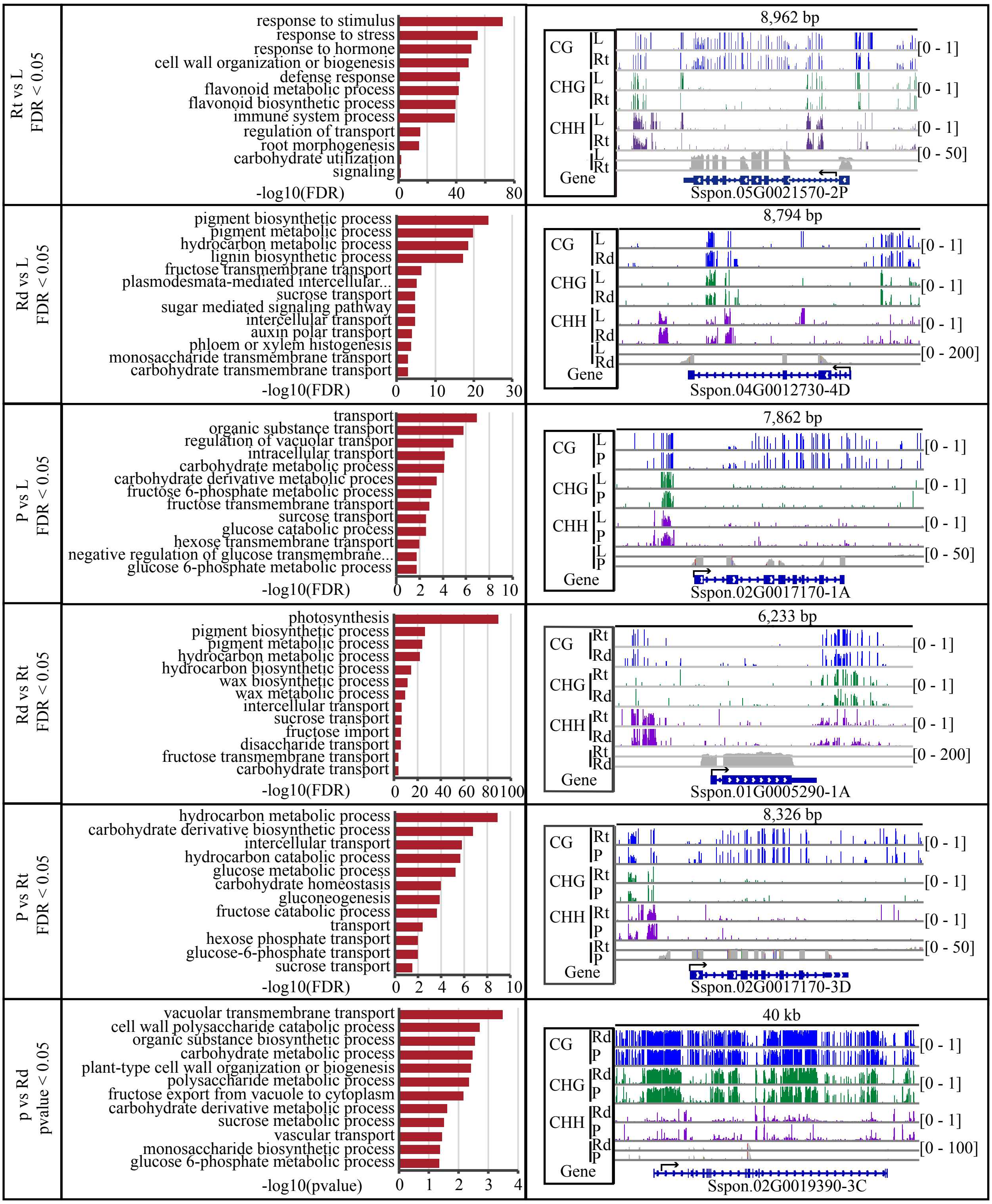

Supplementary Figure 15. GO (the second column) and IGV of Methylation and RNA-seq genome (the third column) browser of up-regulated DEGs with DMR. Blue, green and purple bars indicate CG, CHG, CHH, respectively; gray collapsed bars indicate expression level. L, leaf; Rt, root; Rd, rind; P, pith.

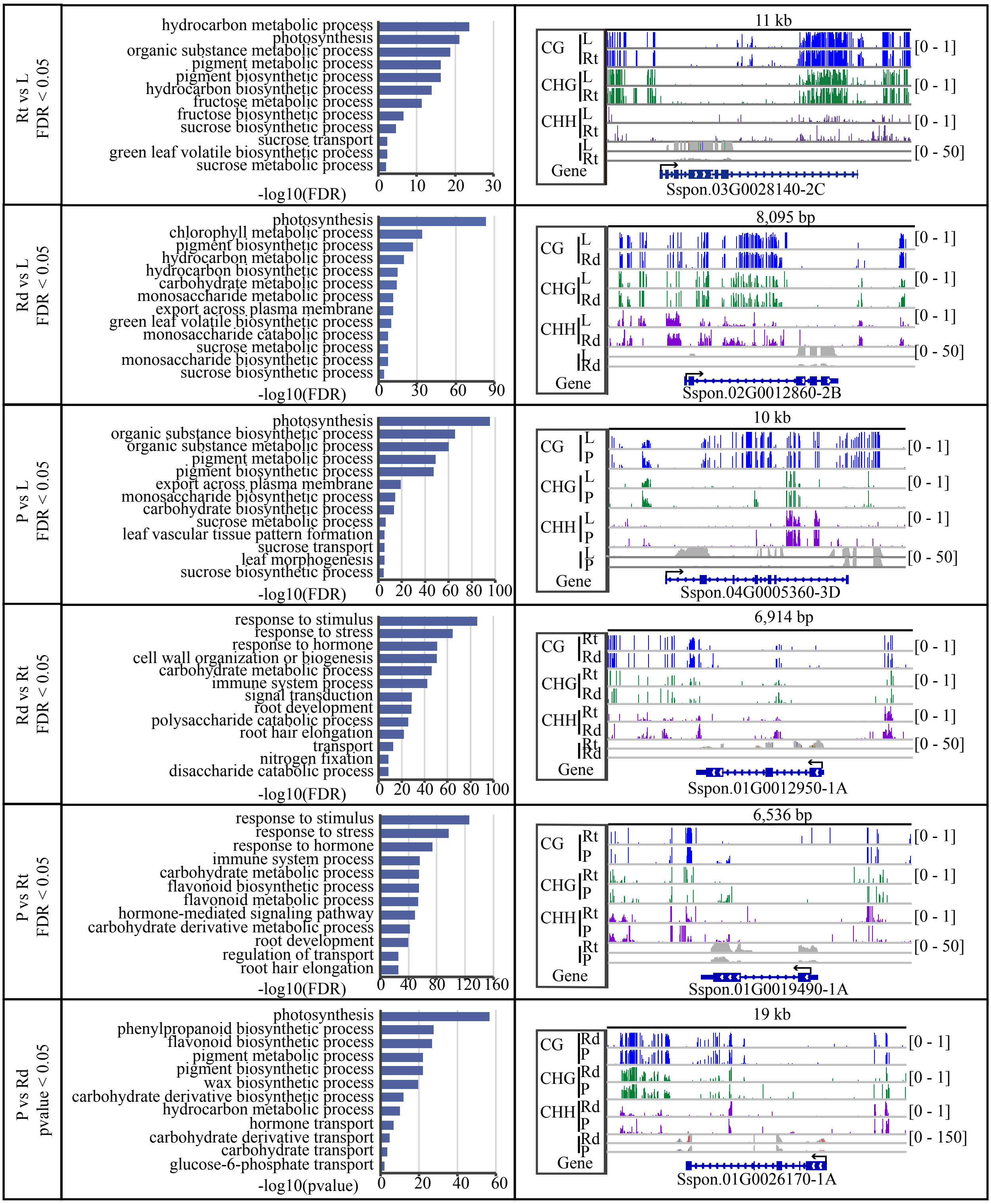

Supplementary Figure 16. GO (the second column) and IGV of Methylation and RNA-seq genome (the third column) browser of down-regulated DEGs with DMR. Blue, green and purple bars indicate CG, CHG, CHH, respectively; gray collapsed bars indicate expression level. L, leaf; Rt, root; Rd, rind; P, pith.

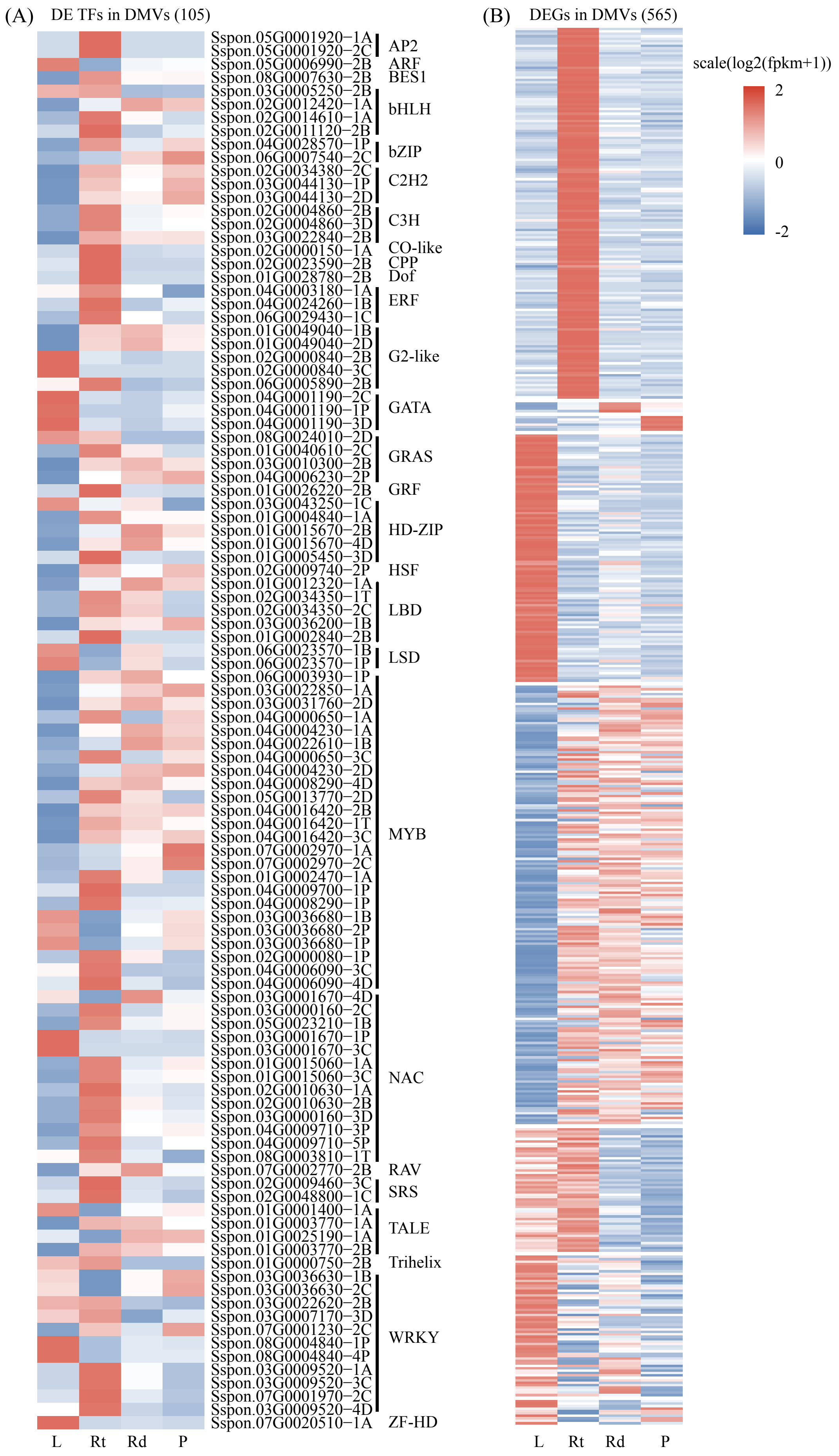

Supplementary Figure 17. The expression patterns of DEGs in DMVs. (A) Heatmap showing the expression patterns of TFs in DMVs; (B) Heatmap showing the expression patterns of genes in DMVs. L, leaf; Rt, root; Rd, rind; P, pith.
